# Supplementary material for: Molecular Insight into Mycobacterium tuberculosis Resistance to Nitrofuranyl Amides Gained through Metagenomics-like Analysis of Spontaneous Mutants
Source: Pharmaceuticals (Basel). 2022 Sep 12;15(9):1136. doi: 10.3390/ph15091136 (PMC9504009; doi:10.3390/ph15091136)

Supplementary Data

Molecular insight into Mycobacterium tuberculosis resistance to nitrofuranyl amides gained through metagenomics-like analysis of spontaneous mutants

Igor Mokrousov [1,7]*, Ivaylo Slavchev [2], Natalia Solovieva [3], Marine Dogonadze [3], Anna Vyazovaya [1], Violeta Valcheva [4], Aleksey Masharsky [5], Olesya Belopolskaya [5], Simeon Dimitrov [4], Viacheslav Zhuravlev [3], Isabel Portugal [6], João Perdigão [6], Georgi M. Dobrikov [2]*

[1] Laboratory of Molecular Epidemiology and Evolutionary Genetics, St Petersburg Pasteur Institute, St Petersburg 197101, Russia

[2] Institute of Organic Chemistry with Centre of Phytochemistry, Bulgarian Academy of Sciences, Acad. G. Bonchev str., bl. 9, Sofia 1113, Bulgaria

[3] St. Petersburg Research Institute of Phthisiopulmonology, St. Petersburg 191036, Russia

[4] The Stephan Angeloff Institute of Microbiology, Bulgarian Academy of Sciences, Acad. G. Bonchev str., bl. 26, Sofia 1113, Bulgaria

[5] Resource Center "Bio-bank Center", Research Park of St. Petersburg State University, St. Petersburg 198504, Russia

[6] iMed.ULisboa – Instituto de Investigação do Medicamento, Faculdade de Farmácia, Universidade de Lisboa, Lisbon, Portugal

[7] Henan International Joint Laboratory of Children's Infectious Diseases, Children's Hospital Affiliated to Zhengzhou University, Zhengzhou 450018, China

* Corresponding authors: Igor Mokrousov, imokrousov@mail.ru
Georgi Dobrikov, georgi.dobrikov@orgchm.bas.bg

1 Chemistry and NMR – general

For thin layer chromatography (TLC) aluminum sheets pre-coated with silica gel 60 F_{254} (Merck) were used. Flash column chromatography was carried out using silica gel 60 (0.040–0.063 mm, 230-400 mesh ASTM, Merck). Commercially available solvents for reactions, TLC and column chromatography were used after distillation (and were dried if needed) – water, petroleum ether (PE), dichloromethane (DCM) and methyl *tert*-butyl ether (MTBE). Any fine chemical, whose synthesis is not described or mentioned in the references in this study, is commercially available from Sigma-Aldrich, Fluorochem, Fluka, Acros or Alfa Aesar. Melting temperatures were determined in capillary tubes on an Electrothermal MEL-TEMP 1102D-230 VAC apparatus without corrections. The NMR spectra were recorded on a Bruker Avance II+ 600 spectrometer (600.13 MHz for ^{1}H and 150.92 MHz for ^{13}C) and Bruker Neo 600 spectrometer (600.18 MHz for ^{1}H and 150.92 MHz for ^{13}C). In case of $CDCl_3$, TMS was used as internal standard (δ=0.00). For other deuterated solvents ^{1}H spectra were calibrated to the residual solvent peaks (DMSO-d_6 δ=2.50). ^{13}C spectra were calibrated in all cases to the residual solvent peaks ($CDCl_3$ δ=77.00, DMSO-d_6 δ=39.52). ^{1}H and ^{13}C NMR data are reported as follows: chemical shift, multiplicity (s = singlet, d = doublet, t = triplet, q = quartet, br = broad, m = multiplet), integration, identification, and coupling constants (in Hz). Singlets in ^{13}C NMR spectra were not mentioned with “s” in all cases. Mass spectra (MS) were recorded on a Shimadzu Liquid Chromatograph Mass Spectrometer LCMS-2020. Direct MS-regime was applied; each compound was dissolved in MeOH with concentration 1 mg/ml; injection volume 3-5 μl; mobile phase – 1% formic acid in MeOH; MS detector with electrospray ionization (ESI). MS spectra are reported as fragmentation in m/z with relative intensities (%). The compound names are in agreement with the IUPAC nomenclature.

1.1 Synthesis of target compounds

1.1.1 Synthesis of *N*-(4-(diethylamino)phenyl)-5-nitrofuran-2-carboxamide (**8**)

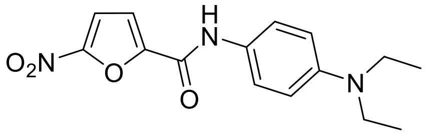

Compound **2** (0.337 g, 2.05 mmol, 1.2 eq.) and Et_3N were dissolved in 20 ml dry DCM. The solution was cooled to 0°C and acyl chloride **1** (0.300 g, 1.71 mmol, 1.0 eq.) was added at once as a solid. The mixture was stirred at r.t. for 18 h (TLC – DCM:MTBE=20:1), then washed with 1% aq. citric acid and water. The organic phase was dried over anhydr. Na_2SO_4 and evaporated. Column chromatography: 1) DCM; 2) DCM:MTBE=20:1 – for product. After column the solid product was washed with hot PE and dried *in vacuo* to give 0.423 g (82%) of pure **8** as red crystals. M.p. 151-152°C. ^{1}H NMR (600.13 MHz, DMSO-d_6, 298 K): δ = 10.36 (br s, 1H, NH), 7.80 (d, 1H, furane $CH_{arom.}$, *J* = 3.9 Hz), 7.56 (d, 1H, furane $CH_{arom.}$, *J* = 3.9 Hz), 7.50 (m, 2H, 2x$CH_{arom.}$), 6.66 (m, 2H, 2x$CH_{arom.}$), 3.32 (q, 4H, 2xCH_2, *J* = 7.0 Hz), 1.01 (t, 6H, 2xCH_3, *J* = 7.0 Hz). ^{13}C NMR (150.92 MHz, DMSO-d_6, 298 K): δ = 153.84 (1C, C=O), 151.61 (1C, quaternary $C_{arom.}$), 148.61 (1C, quaternary $C_{arom.}$), 144.82 (1C, quaternary $C_{arom.}$), 126.08 (1C, quaternary $C_{arom.}$), 122.47 (2C, $CH_{arom.}$), 115.74 (1C, $CH_{arom.}$), 113.63 (1C, $CH_{arom.}$), 111.50 (2C, $CH_{arom.}$), 43.74 (2C, N-CH_2), 12.44 (2C, CH_3). MS (ESI+) m/z (rel. int. %): 304 (M+H, 100), 358 (M+Na+MeOH, 98). (ESI-) m/z (rel. int. %): 302 (M-H, 100). Anal. calcd. for $C_{15}H_{17}N_3O_4$ (303.32): C, 59.40; H, 5.65; N, 13.85. Found: C, 59.47; H, 5.60; N, 13.89 %.

1.1.2 Synthesis of *N*-(2-((1*R*,2*R*,4*S*)-2-hydroxy-1,3,3-trimethylbicyclo[2.2.1]heptan-2-yl)ethyl)-5-nitrofuran-2-carboxamide (**9**)

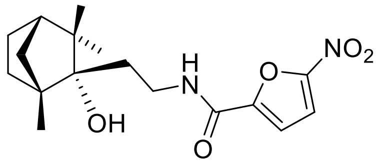

Aminoalcohol **3** (0.340 g, 1.72 mmol, 1.1 eq.) and Et_3N (0.33 ml, 2.35 ml, 1.5 eq.) were dissolved in 20 ml dry DCM and cooled to 5°C. Then acyl chloride **1** was added at once and stirred for 20 h at r.t. Reaction mixture was washed with 1% aq. citric acid and water, dried over anhydr. Na_2SO_4 and evaporated. Column chromatography: 1) DCM; 2) DCM:MTBE = 50:1 – for product. After column the solid product was washed with hot PE and dried *in vacuo* to give pure **9** (0.345 g, 65%) as pale yellow crystals. M.p. 134-135°C. ^{1}H NMR (600.13 MHz, $CDCl_3$, 298 K): δ = 7.36 (d, 1H, furane CH, *J* = 3.8 Hz), 7.30 (apparent br. s, 1H, NH), 7.23 (d, 1H, furane CH, *J* = 3.8 Hz), 3.75 (m, 1H, CH_2CH_2NH), 3.53 (m, 1H, CH_2CH_2NH), 2.08 (br s, 1H, OH), 1.98 (m, 1H, CH_2CH_2NH), 1.75-1.85 (m, 2H, CH_2), 1.71 (m, 1H, CH_2), 1.66 (m, 1H, CH), 1.60 (m, 1H, CH_2), 1.47 (tt, 1H, CH_2, *J* = 12.8, 4.7 Hz), 1.11-1.16 (m, 2H, CH_2), 1.09 (s, CH_3), 1.04 (s, CH_3), 1.02 (s, CH_3). ^{13}C NMR (150.92 MHz, $CDCl_3$, 298 K): δ = 156.10 (1C, C=O), 151.12 (1C, quaternary furane C), 148.41 (1C, quaternary furane C), 115.61 (1C, furane CH), 112.47 (1C, furane CH), 81.80 (1C, quaternary aliphatic C-OH), 52.72 (1C, quaternary aliphatic C), 49.75 (1C, aliphatic CH), 44.21 (1C, quaternary aliphatic C), 40.90 (1C, CH_2), 36.97 (1C, CH_2CH_2NH), 35.43 (1C, CH_2), 30.23 (1C, CH_2), 27.88 (1C, CH_3), 24.78 (1C, CH_2), 22.54 (1C, CH_3), 18.00 (1C, CH_3). MS (ESI+) m/z (rel. int. %): 376 (M+H+K, 72), 391 (M+Na+MeOH, 56). MS (ESI-) m/z (rel. int. %): 335 (M-H, 100), 381 (M+HCOO, 18). Anal. calcd. for $C_{17}H_{24}N_2O_5$ (336.39): C, 60.70; H, 7.19; N, 8.33. Found: C, 60.66; H, 7.24; N, 8.38%.

1.1.3 Synthesis of *N*-(2-(2-methyl-5-nitro-1*H*-imidazol-1-yl)ethyl)-5-nitrofuran-2-carboxamide (**10**)

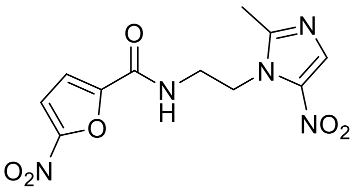

Compound **4** (as dihydrochloride salt, 0.670 g, 2.56 mmol, 1.5 eq.) was suspended in 25 ml dry DCM. Then Et_3N (1.07 ml, 7.70 mmol, 5.4 eq.) and mol. sieve (4Å) were added, cooled to 5°C and stirred for 30 min until clear solution was obtained. Then acyl chloride **1** (0.300 g, 1.71 mmol, 1.0 eq.) was added at once and stirred for 20 h at r.t. Reaction mixture was diluted with 100 ml $CHCl_3$ and filtered through celite pad. The organic phase was washed with water, checked by TLC (DCM:MTBE = 5:1), dried over anhydr. K_2CO_3 and evaporated. The crude product was purified by column chromatography – 1) DCM; 2) DCM:MTBE = 4:1 for product. After column the solid product was washed with hot PE and dried *in vacuo* to give 0.222 g (42%) of pure **10** as pale yellow crystals. M.p. 205-206°C. ^{1}H NMR (600.13 MHz, DMSO-*d*$_6$, 298 K): δ = 9.08 (t, 1H, N<u>H</u>, *J* = 6.1 Hz), 8.04 (s, 1H, imidazole C<u>H</u>), 7.74 (d, 1H, furane C<u>H</u>, *J* = 3.9 Hz), 7.35 (d, 1H, furane C<u>H</u>, *J* = 3.9 Hz), 4.43 (t, 2H, NH-CH_2-C<u>H</u>$_2$, *J* = 5.9 Hz), 3.65 (q, 2H, NH-C<u>H</u>$_2$-CH_2, *J* = 6.1 Hz), 2.38 (s, 3H, C<u>H</u>$_3$). ^{13}C NMR (150.92 MHz, DMSO-*d*$_6$, 298 K): δ = 156.47 (1C, <u>C</u>=O), 151.54 (1C, quaternary $C_{arom.}$), 151.35 (1C, quaternary $C_{arom.}$), 147.64 (1C, quaternary $C_{arom.}$), 138.62 (1C, quaternary $C_{arom.}$), 133.24 (1C, imidazole <u>C</u>H), 115.91 (1C, furane <u>C</u>H), 113.44 (1C, furane <u>C</u>H), 45.16 (1C, NH-CH_2-<u>C</u>H$_2$), 38.27 (1C, NH-<u>C</u>H$_2$-CH_2), 13.81 (1C, <u>C</u>H$_3$). MS (ESI+) m/z (rel. int. %): 364 (M+Na+MeOH, 100), 310 (M+H, 26). (ESI-) m/z (rel. int. %): 308 (M-H, 100). Anal. calcd. for $C_{11}H_{11}N_5O_6$ (309.24): C, 42.72; H, 3.59; N, 22.65. Found: C, 42.79; H, 3.64; N, 22.60%.

1.1.4 Synthesis of (4-(3-hydroxyphenyl)piperazin-1-yl)(5-nitrofuran-2-yl)methanone (**11**)

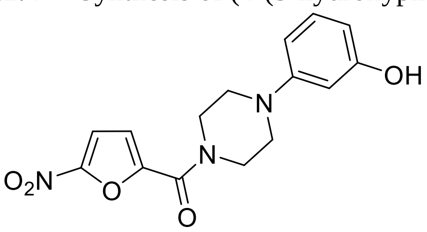

Compound **5** (0.384 g, 2.05 mmol, 1.2 eq.) was suspended in 25 ml dry DCM. Et_3N (0.54 ml, 3.85 mmol, 2.3 eq.) was added and cooled to 5°C. Then acylchloride **1** (0.300 g, 1.71 mmol, 1.0 eq.) was added at once and stirred for 48 h at r.t. Reaction mixture was diluted with DCM and filtered through celite pad. The filtrate was washed with water, dried over anhydr. Na_2SO_4 and evaporated. Crude product was purified by column chromatography – 1) DCM; 2) DCM:MTBE = 10:1 – for product. The solid product was washed with PE and dried in vacuo to give 0.135 g (25%) of pure **11** as pale yellow crystals. M.p. 179-180°C. ^{1}H NMR (600.18 MHz, DMSO-*d*$_6$, 353 K): δ = 8.89 (s, 1H, O<u>H</u>), 7.69 (d, 1H, furane C<u>H</u>, *J* = 3.8 Hz), 7.26 (d, 1H, furane C<u>H</u>, *J* = 3.8 Hz), 7.01 (t, 1H, aromatic C<u>H</u>, *J* = 8.1 Hz), 6.41 (m, 1H, aromatic C<u>H</u>), 6.36 (t, 1H, aromatic C<u>H</u>, *J* = 2.3 Hz), 6.27 (m, 1H, aromatic C<u>H</u>), 3.81 (m, 4H, piperazine 2xC<u>H</u>$_2$), 3.21 (m, 4H, piperazine 2xC<u>H</u>$_2$). ^{13}C NMR (150.92 MHz, DMSO-*d*$_6$, 353 K): δ = 157.85 (1C, <u>C</u>=O), 156.37 (1C, quaternary $C_{arom.}$), 151.50 (1C, quaternary $C_{arom.}$), 150.85 (1C, quaternary $C_{arom.}$), 147.51 (1C, quaternary $C_{arom.}$), 129.15 (1C, aromatic <u>C</u>H), 116.48 (1C, furane <u>C</u>H), 112.23 (1C, furane <u>C</u>H), 106.58 (1C, aromatic <u>C</u>H), 106.51 (1C, aromatic <u>C</u>H), 102.71 (1C, aromatic <u>C</u>H), 48.04 (2C, piperazine 2x<u>C</u>H$_2$), 43.74 (br s, 2C, piperazine 2x<u>C</u>H$_2$). MS (ESI+) m/z (rel. int. %): 318 (M+H, 16), 340 (M+Na, 98), 372 (M+Na+MeOH, 100). MS (ESI-) m/z (rel. int. %): 316 (M-H, 100), 362 (M+HCOO, 73), 633 (2M-H, 52). Anal. calcd. for $C_{15}H_{15}N_3O_5$ (317.30): C, 56.78; H, 4.77; N, 13.24. Found: C, 56.83; H, 4.79; N, 13.20%.

1.1.5 Synthesis of (5-nitrofuran-2-yl)(4-(pyridin-2-yl)piperazin-1-yl)methanone (**12**)

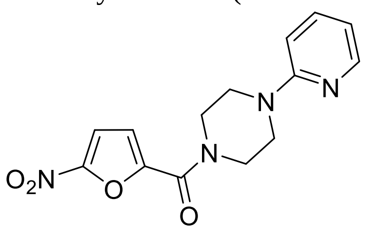

Compound **6** (0.334 g, 2.05 mmol, 1.15 eq.) was dissolved in 25 ml dry DCM. Et_3N (0.54 ml, 3.85 mmol, 2.1 eq.) was added and cooled to 5°C. Then acylchloride **1** (0.317 g, 1.81 mmol, 1.0 eq.) was added at once and stirred for 18 h at r.t. Reaction mixture was washed with water, dried over anhydr. Na_2SO_4 and evaporated. Crude product was purified by column chromatography – DCM:MTBE = 10:1. The solid product was washed with PE and dried *in vacuo* to give 0.411 g (75%) of pure **12** as yellow crystals. M.p. 149-150°C. ^{1}H NMR (600.18 MHz, DMSO-d_6, 353 K): δ = 8.14 (m, 1H, aromatic CH), 7.70 (d, 1H, furane CH, *J* = 3.9 Hz), 7.56 (ddd, 1H, aromatic CH, *J* = 8.9, 7.1, 2.0 Hz), 7.27 (d, 1H, furane CH, *J* = 3.9 Hz), 6.83 (m, 1H, aromatic CH), 6.67 (dd, 1H, aromatic CH, *J* = 7.1, 4.9 Hz), 3.80 (m, 4H, piperazine 2xCH_2), 3.64 (m, 4H, piperazine 2xCH_2). ^{13}C NMR (150.92 MHz, DMSO-d_6, 353 K): δ = 158.24 (1C, C=O), 156.51 (1C, quaternary $C_{arom.}$), 150.85 (1C, quaternary $C_{arom.}$), 147.51 (1C, quaternary $C_{arom.}$), 147.18 (1C, aromatic CH), 137.13 (1C, aromatic CH), 116.49 (1C, aromatic CH), 112.92 (1C, aromatic CH), 112.22 (1C, aromatic CH), 106.84 (1C, aromatic CH), 44.14 (2C, piperazine 2xCH_2), 43.70 (br s, 2C, piperazine 2xCH_2). MS (ESI+) m/z (rel. int. %): 303 (M+H, 70), 357 (M+Na+MeOH, 100). MS (ESI-) m/z (rel. int. %): 285 (M-H-O, 100). Anal. calcd. for $C_{14}H_{14}N_4O_4$ (302.29): C, 55.63; H, 4.67; N, 18.53. Found: C, 55.68; H, 4.61; N, 18.57%.

1.1.6 Synthesis of (5-nitrofuran-2-yl)(4-(4-nitrophenyl)piperazin-1-yl)methanone (**13**)

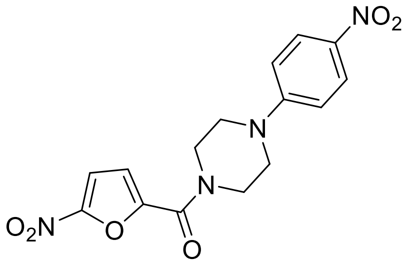

Compound **7** (0.425 g, 2.05 mmol, 1.15 eq.) was dissolved in 25 ml dry DCM. Et_3N (0.36 ml, 2.56 mmol, 1.5 eq.) was added and cooled to 5°C. Then acylchloride **1** (0.300 g, 1.71 mmol, 1.0 eq.) was added at once and stirred for 18 h at r.t. Reaction mixture was washed with water, dried over anhydr. Na_2SO_4 and evaporated. Crude product was purified by column chromatography – DCM:MTBE = 50:1. The solid product was washed with PE and dried *in vacuo* to give 0.540 g (91%) of pure **13** as bright orange crystals. M.p. 200-201°C. ^{1}H NMR (600.18 MHz, DMSO-d_6, 353 K): δ = 8.08 (m, 2H, phenyl aromatic CH), 7.70 (d, 1H, furane CH, *J* = 3.9 Hz), 7.29 (d, 1H, furane CH, *J* = 3.9 Hz), 7.02 (m, 2H, phenyl aromatic CH), 3.88 (m, 4H, piperazine 2xCH_2), 3.64 (m, 4H, piperazine 2xCH_2). ^{13}C NMR (150.92 MHz, DMSO-d_6, 353 K): δ = 156.53 (1C, C=O), 153.86 (1C, quaternary $C_{arom.}$), 150.88 (1C, quaternary $C_{arom.}$), 147.35 (1C, quaternary $C_{arom.}$), 137.20 (1C, aromatic CH), 125.17 (2C, phenyl aromatic CH), 116.78 (1C, aromatic CH), 112.24 (3C, furane CH and 2 phenyl aromatic CH), 45.56 (2C, piperazine 2xCH_2), 43.26 (br s, 2C, piperazine 2xCH_2). MS (ESI+) m/z (rel. int. %): 369 (M+Na, 31), 385 (M+K, 28), 401 (M+Na+MeOH, 100). MS (ESI-) m/z (rel. int. %): 391 (M+HCOO, 100). Anal. calcd. for $C_{15}H_{14}N_4O_6$ (346.30): C, 52.03; H, 4.08; N, 16.18. Found: C, 51.97; H, 4.10; N, 16.14%.

2. ^{1}H and ^{13}C NMR spectra of synthesized compounds

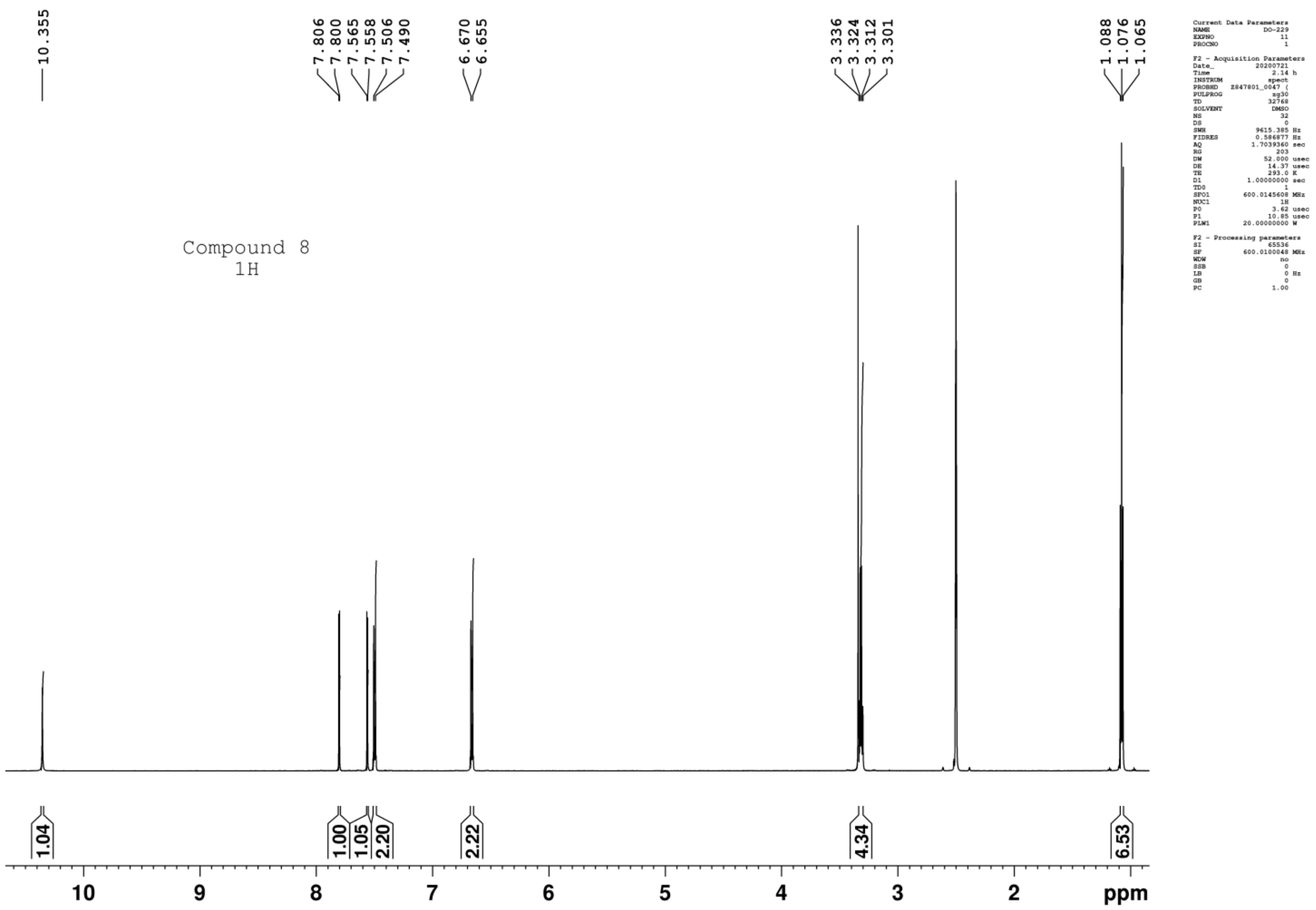
10.355
7.806
7.800
7.565
7.558
7.506
7.490
6.670
6.655
3.336
3.324
3.312
3.301
1.088
1.076
1.065
Compound 8
1H
1.04
1.00
1.05
2.20
2.22
4.34
6.53
10
9
8
7
6
5
4
3
2
ppm
Current Data Parameters
NAME DO-229
EXPNO 11
PROCNO 1
F2 - Acquisition Parameters
Date_ 20200721
Time 2.14 h
INSTRUM spect
PROBHD Z847801_0047 (
PULPROG zg30
TD 32768
SOLVENT DMSO
NS 32
DS 0
SWH 9615.385 Hz
FIDRES 0.586877 Hz
AQ 1.7039360 sec
RG 203
DW 52.000 usec
DE 14.37 usec
TE 293.0 K
D1 1.00000000 sec
TD0 1
SFO1 600.0145608 MHz
NUC1 1H
P0 3.62 usec
P1 10.85 usec
PLW1 20.00000000 W
F2 - Processing parameters
SI 65536
SF 600.0100048 MHz
WDW no
SSB 0
LB 0 Hz
GB 0
PC 1.00

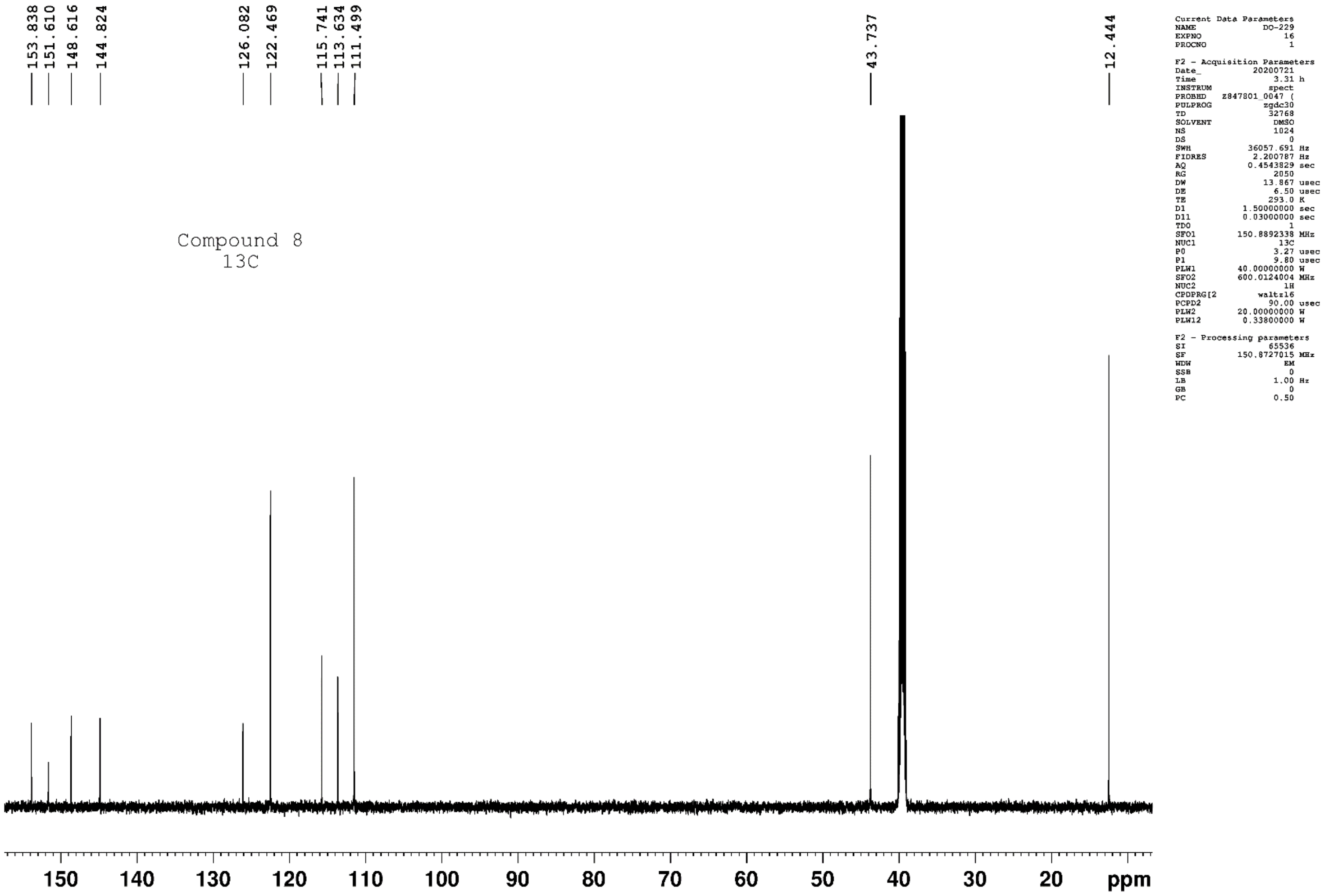
153.838
151.610
148.616
144.824
126.082
122.469
115.741
113.634
111.499
43.737
12.444
Compound 8
13C
Current Data Parameters
NAME DO-229
EXPNO 16
PROCNO 1
F2 - Acquisition Parameters
Date_ 20200721
Time 3.31 h
INSTRUM spect
PROBHD Z847801_0047 (
PULPROG zgdc30
TD 32768
SOLVENT DMSO
NS 1024
DS 0
SWH 36057.691 Hz
FIDRES 2.200787 Hz
AQ 0.4543829 sec
RG 2050
DW 13.867 usec
DE 6.50 usec
TE 293.0 K
D1 1.50000000 sec
D11 0.03000000 sec
TD0 1
SFO1 150.8892338 MHz
NUC1 13C
P0 3.27 usec
P1 9.80 usec
PLW1 40.00000000 W
SFO2 600.0124004 MHz
NUC2 1H
CPDPRG[2 waltz16
PCPD2 90.00 usec
PLW2 20.00000000 W
PLW12 0.33800000 W
F2 - Processing parameters
SI 65536
SF 150.8727015 MHz
WDW EM
SSB 0
LB 1.00 Hz
GB 0
PC 0.50
150
140
130
120
110
100
90
80
70
60
50
40
30
20
ppm

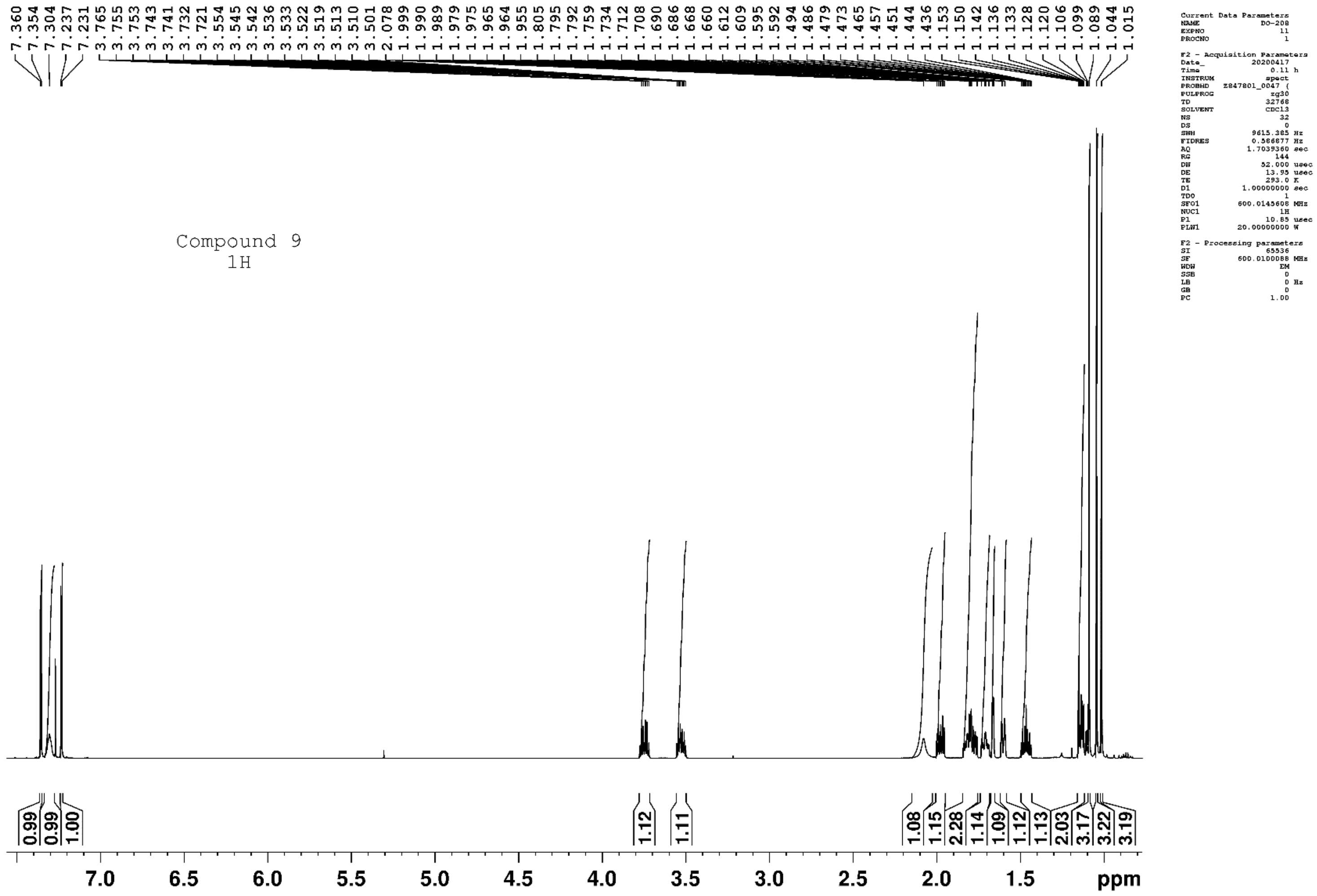
Compound 9
1H
Current Data Parameters
NAME DO-208
EXPNO 11
PROCNO 1
ppm

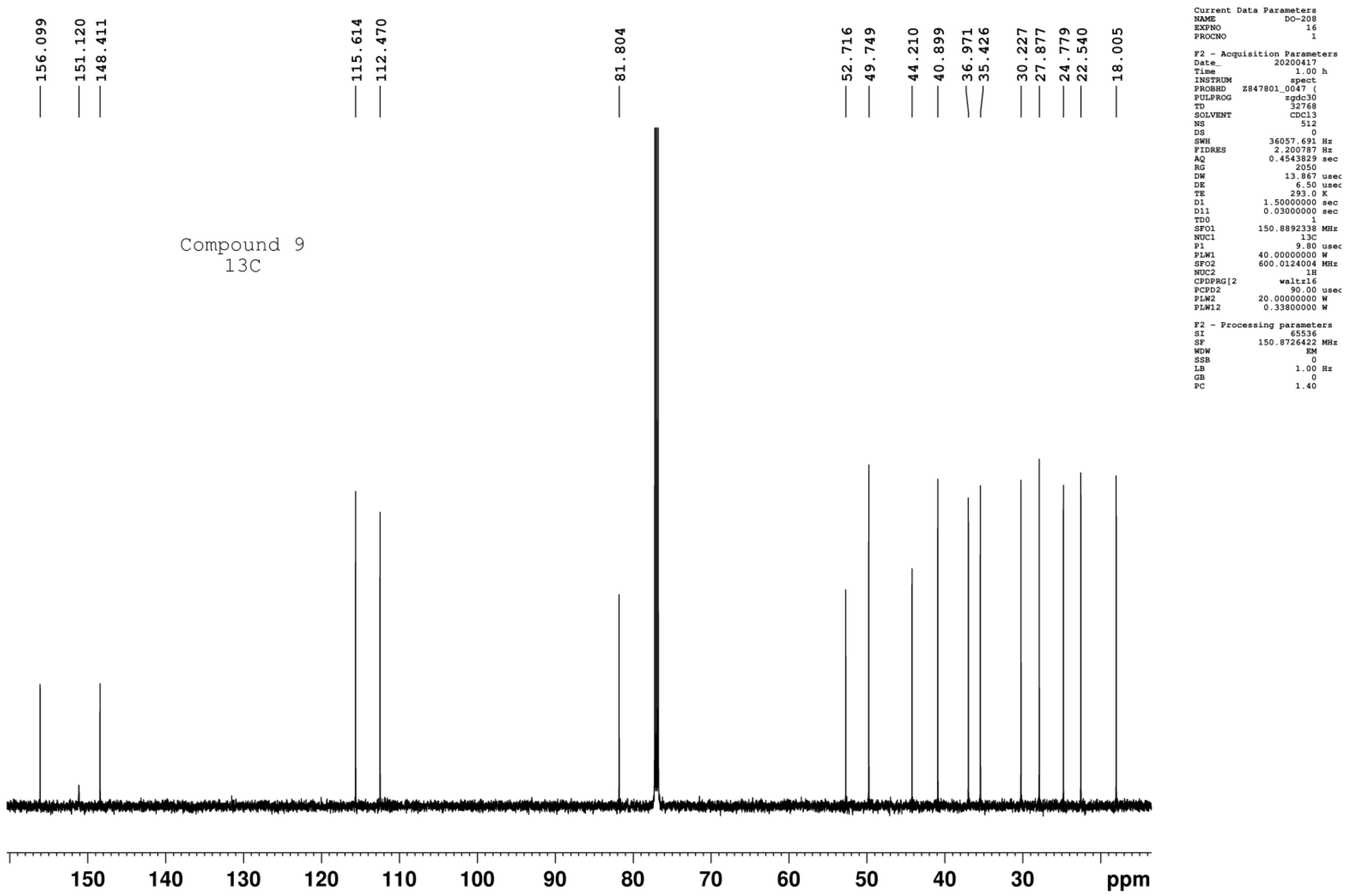
156.099
151.120
148.411
115.614
112.470
81.804
52.716
49.749
44.210
40.899
36.971
35.426
30.227
27.877
24.779
22.540
18.005
Compound 9
13C
150
140
130
120
110
100
90
80
70
60
50
40
30
ppm
Current Data Parameters
NAME DO-208
EXPNO 16
PROCNO 1
F2 - Acquisition Parameters
Date_ 20200417
Time 1.00 h
INSTRUM spect
PROBHD Z847801_0047 (
PULPROG zgdc30
TD 32768
SOLVENT CDCl3
NS 512
DS 0
SWH 36057.691 Hz
FIDRES 2.200787 Hz
AQ 0.4543829 sec
RG 2050
DW 13.867 usec
DE 6.50 usec
TE 293.0 K
D1 1.50000000 sec
D11 0.03000000 sec
TD0 1
SFO1 150.8892338 MHz
NUC1 13C
P1 9.80 usec
PLW1 40.00000000 W
SFO2 600.0124004 MHz
NUC2 1H
CPDPRG[2 waltz16
PCPD2 90.00 usec
PLW2 20.00000000 W
PLW12 0.33800000 W
F2 - Processing parameters
SI 65536
SF 150.8726422 MHz
WDW EM
SSB 0
LB 1.00 Hz
GB 0
PC 1.40

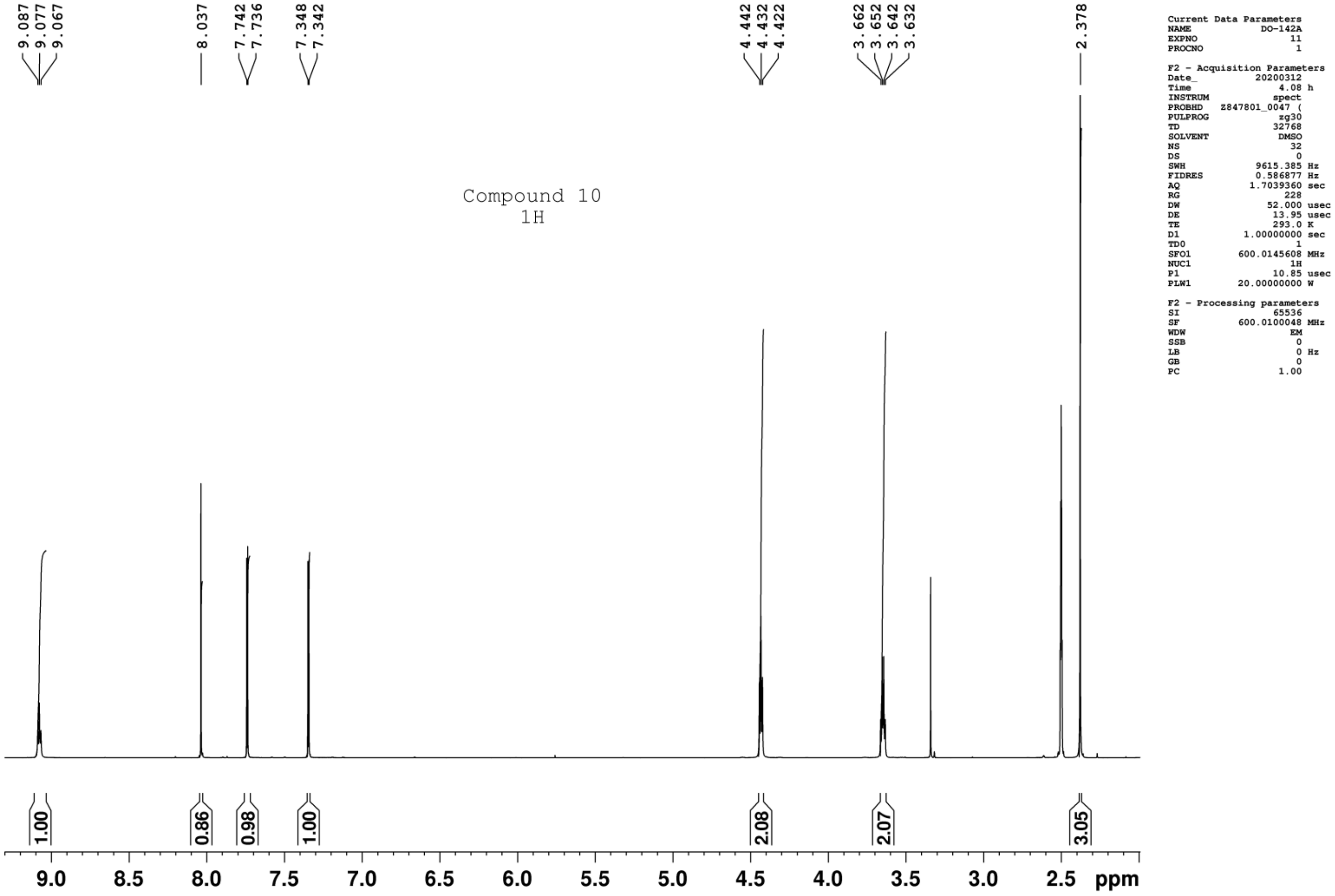

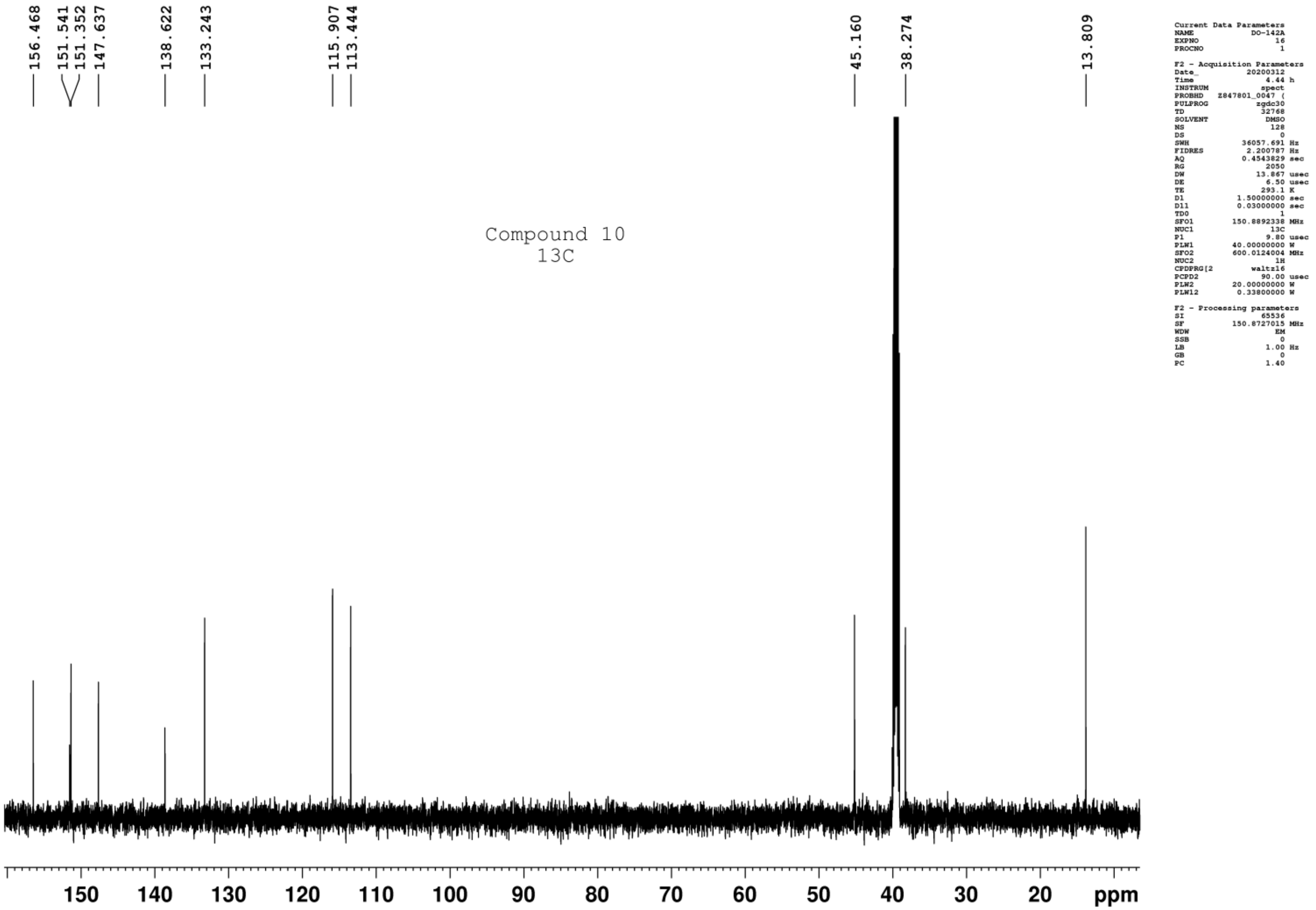
156.468
151.541
151.352
147.637
138.622
133.243
115.907
113.444
45.160
38.274
13.809
Compound 10
13C
150
140
130
120
110
100
90
80
70
60
50
40
30
20
ppm
Current Data Parameters
NAME DO-142A
EXPNO 16
PROCNO 1
F2 - Acquisition Parameters
Date_ 20200312
Time 4.44 h
INSTRUM spect
PROBHD Z847801_0047 (
PULPROG zgdc30
TD 32768
SOLVENT DMSO
NS 128
DS 0
SWH 36057.691 Hz
FIDRES 2.200787 Hz
AQ 0.4543829 sec
RG 2050
DW 13.867 usec
DE 6.50 usec
TE 293.1 K
D1 1.50000000 sec
D11 0.03000000 sec
TD0 1
SFO1 150.8892338 MHz
NUC1 13C
P1 9.80 usec
PLW1 40.00000000 W
SFO2 600.0124004 MHz
NUC2 1H
CPDPRG[2 waltz16
PCPD2 90.00 usec
PLW2 20.00000000 W
PLW12 0.33800000 W
F2 - Processing parameters
SI 65536
SF 150.8727015 MHz
WDW EM
SSB 0
LB 1.00 Hz
GB 0
PC 1.40

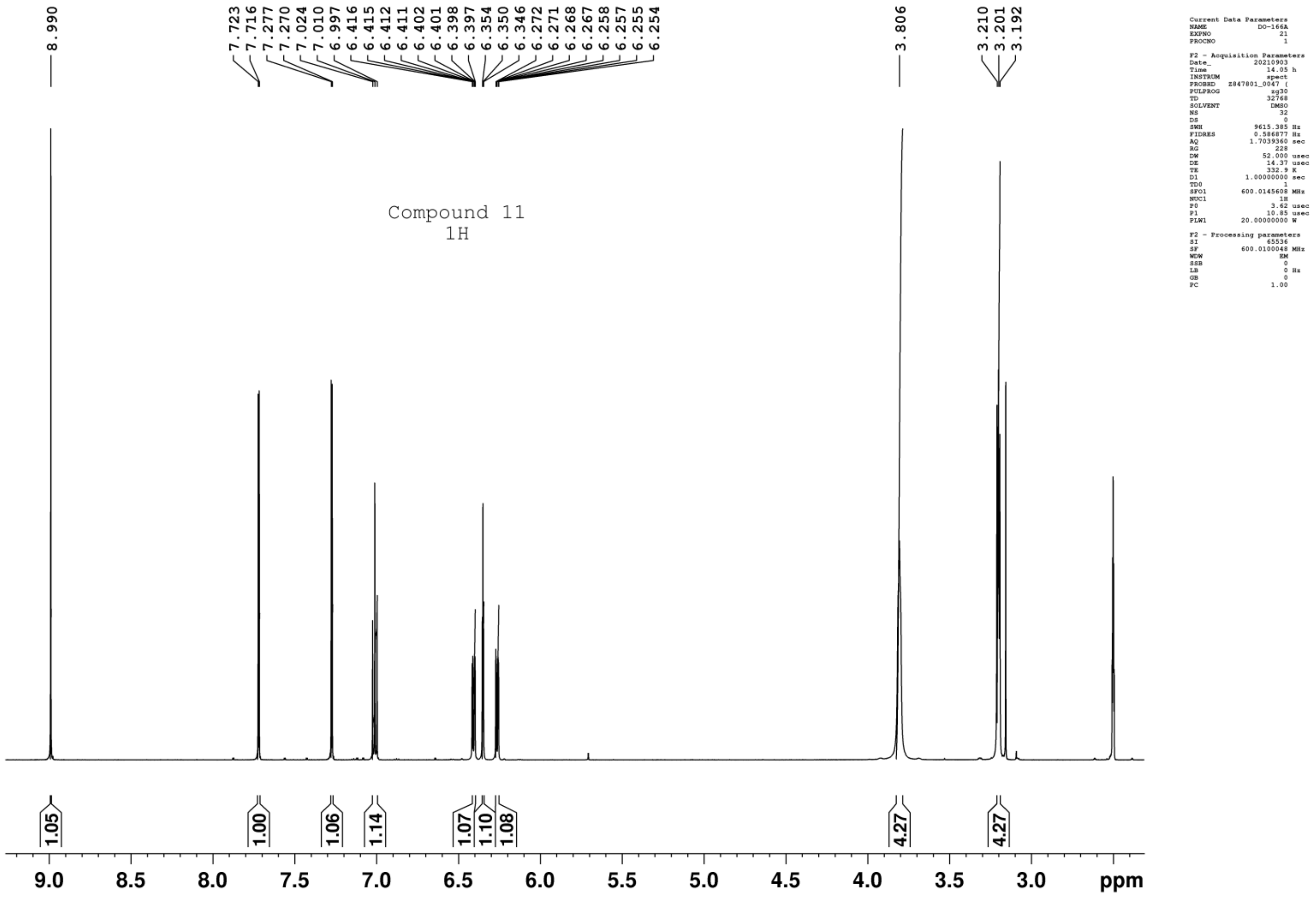
8.990
7.723
7.716
7.277
7.270
7.024
7.010
6.997
6.416
6.415
6.412
6.411
6.402
6.401
6.398
6.397
6.354
6.350
6.346
6.272
6.271
6.268
6.267
6.258
6.257
6.255
6.254
3.806
3.210
3.201
3.192
Compound 11
1H
1.05
1.00
1.06
1.14
1.07
1.10
1.08
4.27
4.27
9.0
8.5
8.0
7.5
7.0
6.5
6.0
5.5
5.0
4.5
4.0
3.5
3.0
ppm
Current Data Parameters
NAME DO-166A
EXPNO 21
PROCNO 1
F2 - Acquisition Parameters
Date_ 20210903
Time 14.05 h
INSTRUM spect
PROBHD Z847801_0047 (
PULPROG zg30
TD 32768
SOLVENT DMSO
NS 32
DS 0
SWH 9615.385 Hz
FIDRES 0.586877 Hz
AQ 1.7039360 sec
RG 228
DW 52.000 usec
DE 14.37 usec
TE 332.9 K
D1 1.00000000 sec
TD0 1
SFO1 600.0145608 MHz
NUC1 1H
P0 3.62 usec
P1 10.85 usec
PLW1 20.00000000 W
F2 - Processing parameters
SI 65536
SF 600.0100048 MHz
WDW EM
SSB 0
LB 0 Hz
GB 0
PC 1.00

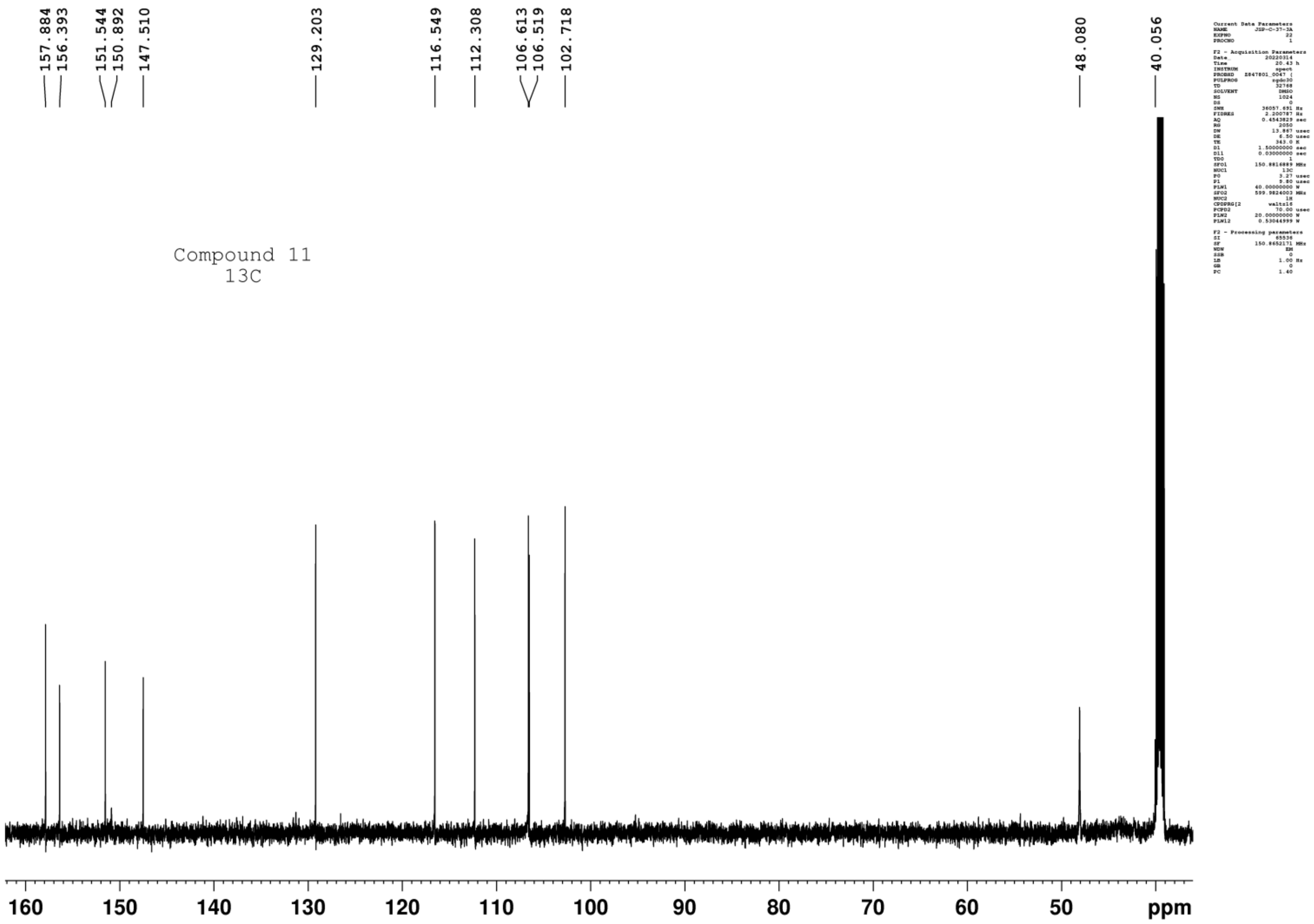
157.884
156.393
151.544
150.892
147.510
129.203
116.549
112.308
106.613
106.519
102.718
48.080
40.056
Compound 11
13C
160
150
140
130
120
110
100
90
80
70
60
50
ppm

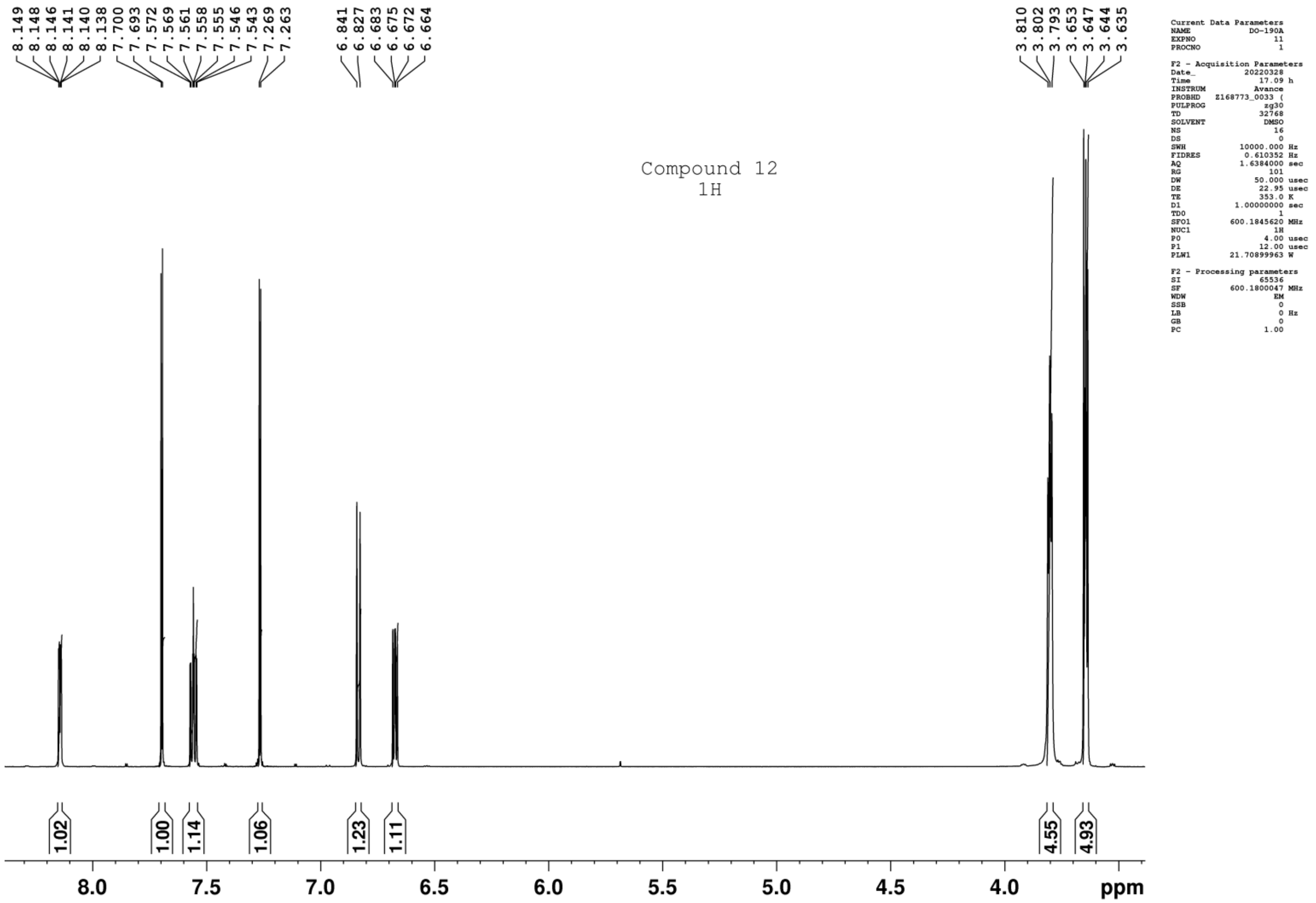
8.149
8.148
8.146
8.141
8.140
8.138
7.700
7.693
7.572
7.569
7.561
7.558
7.555
7.546
7.543
7.269
7.263
6.841
6.827
6.683
6.675
6.672
6.664
3.810
3.802
3.793
3.653
3.647
3.644
3.635
Compound 12
1H
1.02
1.00
1.14
1.06
1.23
1.11
4.55
4.93
8.0
7.5
7.0
6.5
6.0
5.5
5.0
4.5
4.0
ppm
Current Data Parameters
NAME DO-190A
EXPNO 11
PROCNO 1
F2 - Acquisition Parameters
Date_ 20220328
Time 17.09 h
INSTRUM Avance
PROBHD Z168773_0033 (
PULPROG zg30
TD 32768
SOLVENT DMSO
NS 16
DS 0
SWH 10000.000 Hz
FIDRES 0.610352 Hz
AQ 1.6384000 sec
RG 101
DW 50.000 usec
DE 22.95 usec
TE 353.0 K
D1 1.00000000 sec
TD0 1
SFO1 600.1845620 MHz
NUC1 1H
P0 4.00 usec
P1 12.00 usec
PLW1 21.70899963 W
F2 - Processing parameters
SI 65536
SF 600.1800047 MHz
WDW EM
SSB 0
LB 0 Hz
GB 0
PC 1.00

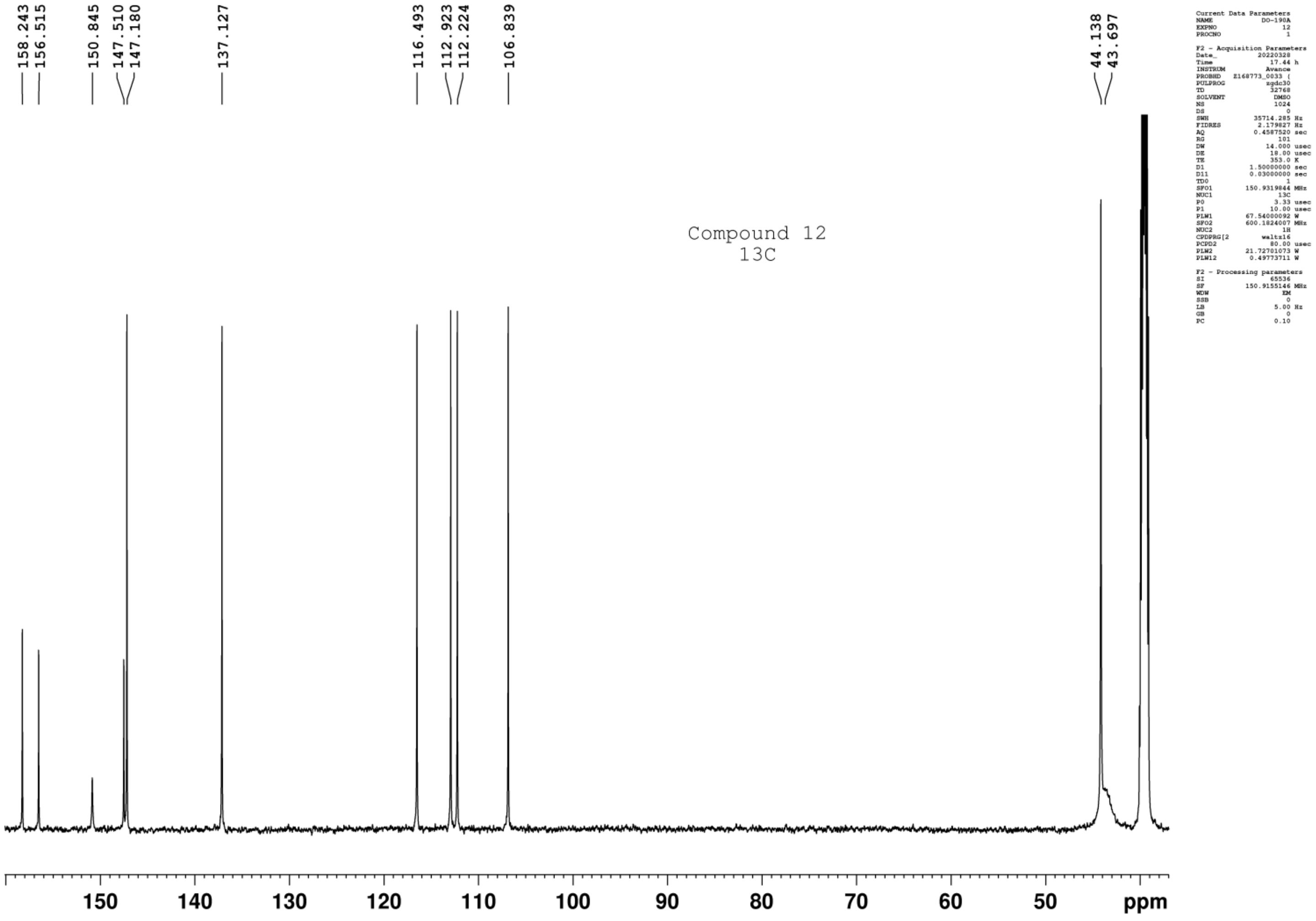
158.243
156.515
150.845
147.510
147.180
137.127
116.493
112.923
112.224
106.839
44.138
43.697
Compound 12
13C
Current Data Parameters
NAME DO-190A
EXPNO 12
PROCNO 1
F2 - Acquisition Parameters
Date_ 20220328
Time 17.44 h
INSTRUM Avance
PROBHD Z168773_0033 (
PULPROG zgdc30
TD 32768
SOLVENT DMSO
NS 1024
DS 0
SWH 35714.285 Hz
FIDRES 2.179827 Hz
AQ 0.4587520 sec
RG 101
DW 14.000 usec
DE 18.00 usec
TE 353.0 K
D1 1.50000000 sec
D11 0.03000000 sec
TD0 1
SFO1 150.9319844 MHz
NUC1 13C
P0 3.33 usec
P1 10.00 usec
PLW1 67.54000092 W
SFO2 600.1824007 MHz
NUC2 1H
CPDPRG[2 waltz16
PCPD2 80.00 usec
PLW2 21.72701073 W
PLW12 0.49773711 W
F2 - Processing parameters
SI 65536
SF 150.9155146 MHz
WDW EM
SSB 0
LB 5.00 Hz
GB 0
PC 0.10
150
140
130
120
110
100
90
80
70
60
50
ppm

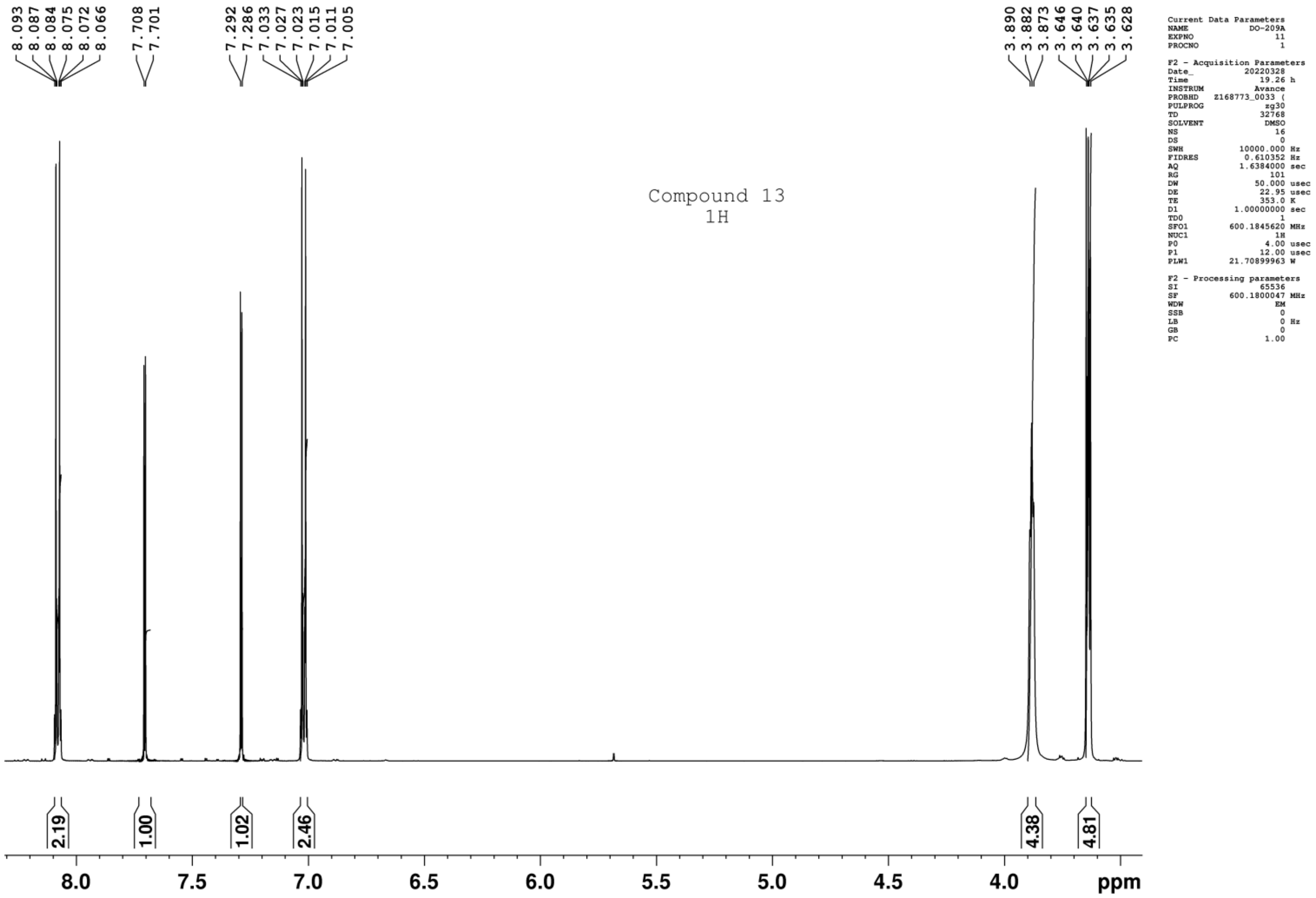
8.093
8.087
8.084
8.075
8.072
8.066
7.708
7.701
7.292
7.286
7.033
7.027
7.023
7.015
7.011
7.005
3.890
3.882
3.873
3.646
3.640
3.637
3.635
3.628
Compound 13
1H
2.19
1.00
1.02
2.46
4.38
4.81
8.0
7.5
7.0
6.5
6.0
5.5
5.0
4.5
4.0
ppm
Current Data Parameters
NAME DO-209A
EXPNO 11
PROCNO 1
F2 - Acquisition Parameters
Date_ 20220328
Time 19.26 h
INSTRUM Avance
PROBHD Z168773_0033 (
PULPROG zg30
TD 32768
SOLVENT DMSO
NS 16
DS 0
SWH 10000.000 Hz
FIDRES 0.610352 Hz
AQ 1.6384000 sec
RG 101
DW 50.000 usec
DE 22.95 usec
TE 353.0 K
D1 1.00000000 sec
TD0 1
SFO1 600.1845620 MHz
NUC1 1H
P0 4.00 usec
P1 12.00 usec
PLW1 21.70899963 W
F2 - Processing parameters
SI 65536
SF 600.1800047 MHz
WDW EM
SSB 0
LB 0 Hz
GB 0
PC 1.00

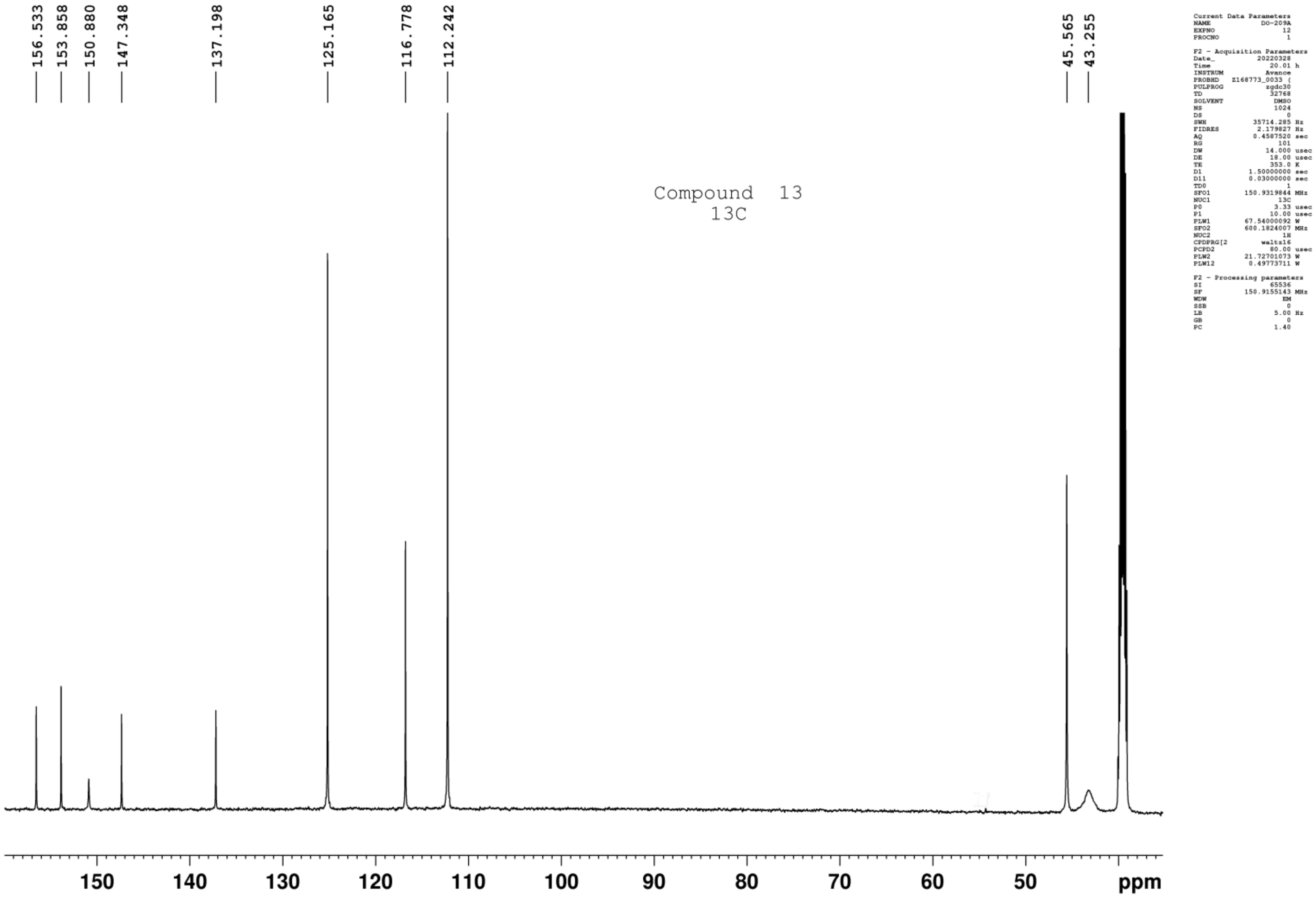
156.533
153.858
150.880
147.348
137.198
125.165
116.778
112.242
45.565
43.255
Compound 13
13C
150
140
130
120
110
100
90
80
70
60
50
ppm
Current Data Parameters
NAME DO-209A
EXPNO 12
PROCNO 1
F2 - Acquisition Parameters
Date_ 20220328
Time 20.01 h
INSTRUM Avance
PROBHD Z168773_0033 (
PULPROG zgdc30
TD 32768
SOLVENT DMSO
NS 1024
DS 0
SWH 35714.285 Hz
FIDRES 2.179827 Hz
AQ 0.4587520 sec
RG 101
DW 14.000 usec
DE 18.00 usec
TE 353.0 K
D1 1.50000000 sec
D11 0.03000000 sec
TD0 1
SFO1 150.9319844 MHz
NUC1 13C
P0 3.33 usec
P1 10.00 usec
PLW1 67.54000092 W
SFO2 600.1824007 MHz
NUC2 1H
CPDPRG[2 waltz16
PCPD2 80.00 usec
PLW2 21.72701073 W
PLW12 0.49773711 W
F2 - Processing parameters
SI 65536
SF 150.9155143 MHz
WDW EM
SSB 0
LB 5.00 Hz
GB 0
PC 1.40

3. MS spectra of synthesized compounds

==== Shimadzu LabSolutions Browser Report ====

MS Spectrum(DO-229.lcd)

Compound 8

Ret. Time: 1-1(E+) 1-2(E-) [0.193->0.633]-[0.073->0.960]

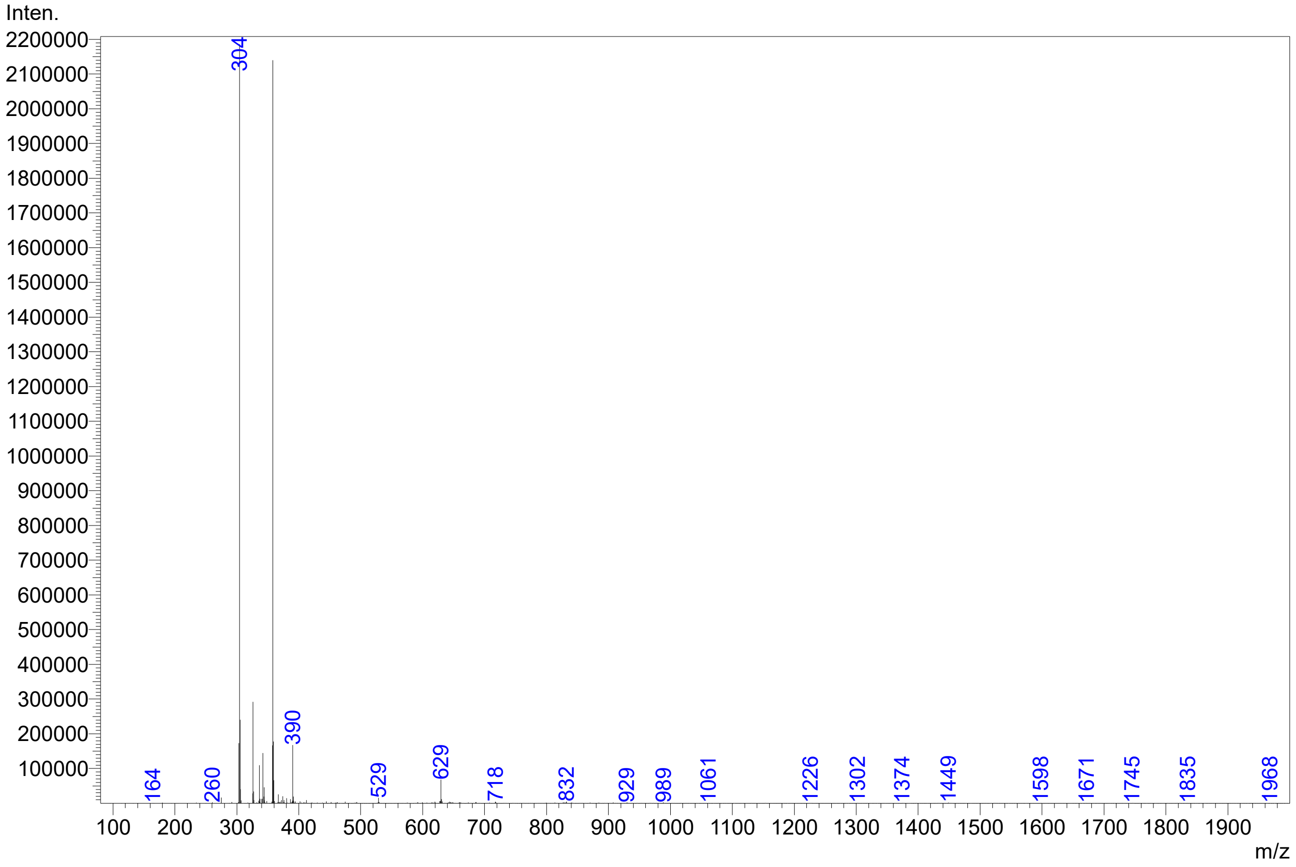

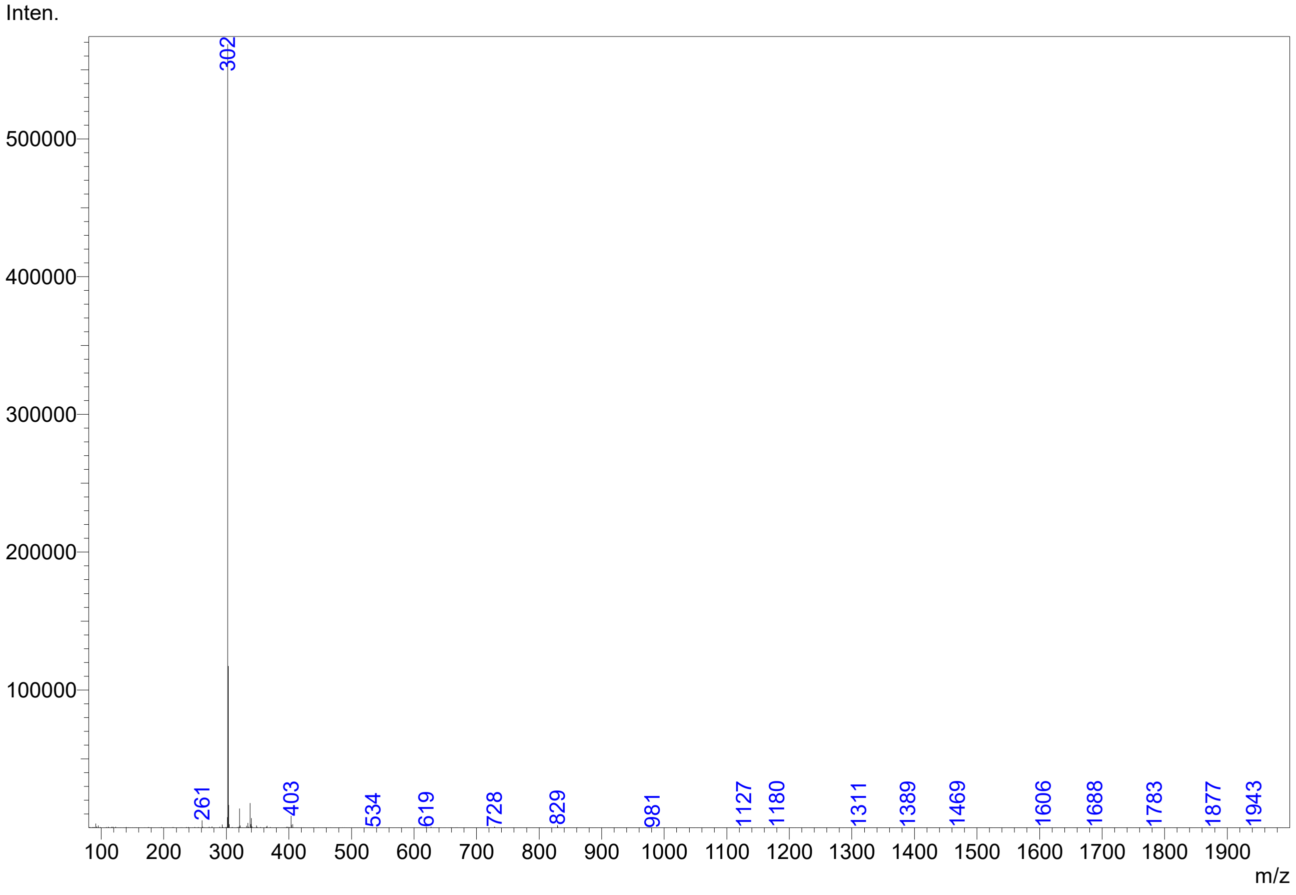

==== Shimadzu LabSolutions Browser Report ====

MS Spectrum(DO-208.lcd)

Ret. Time: 1-1(E+) 1-2(E-) [0.193->0.720]-[0.120->1.013]

Compound 9

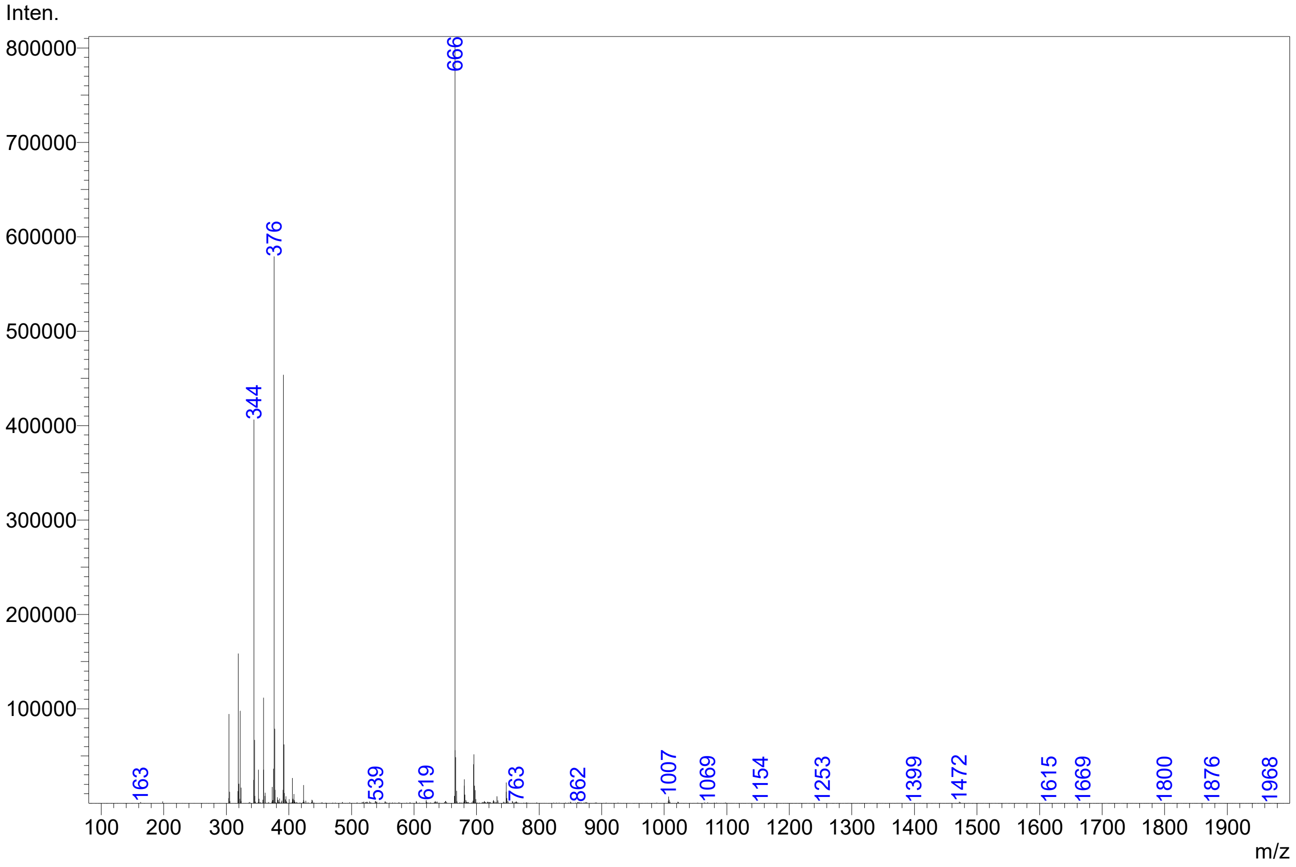

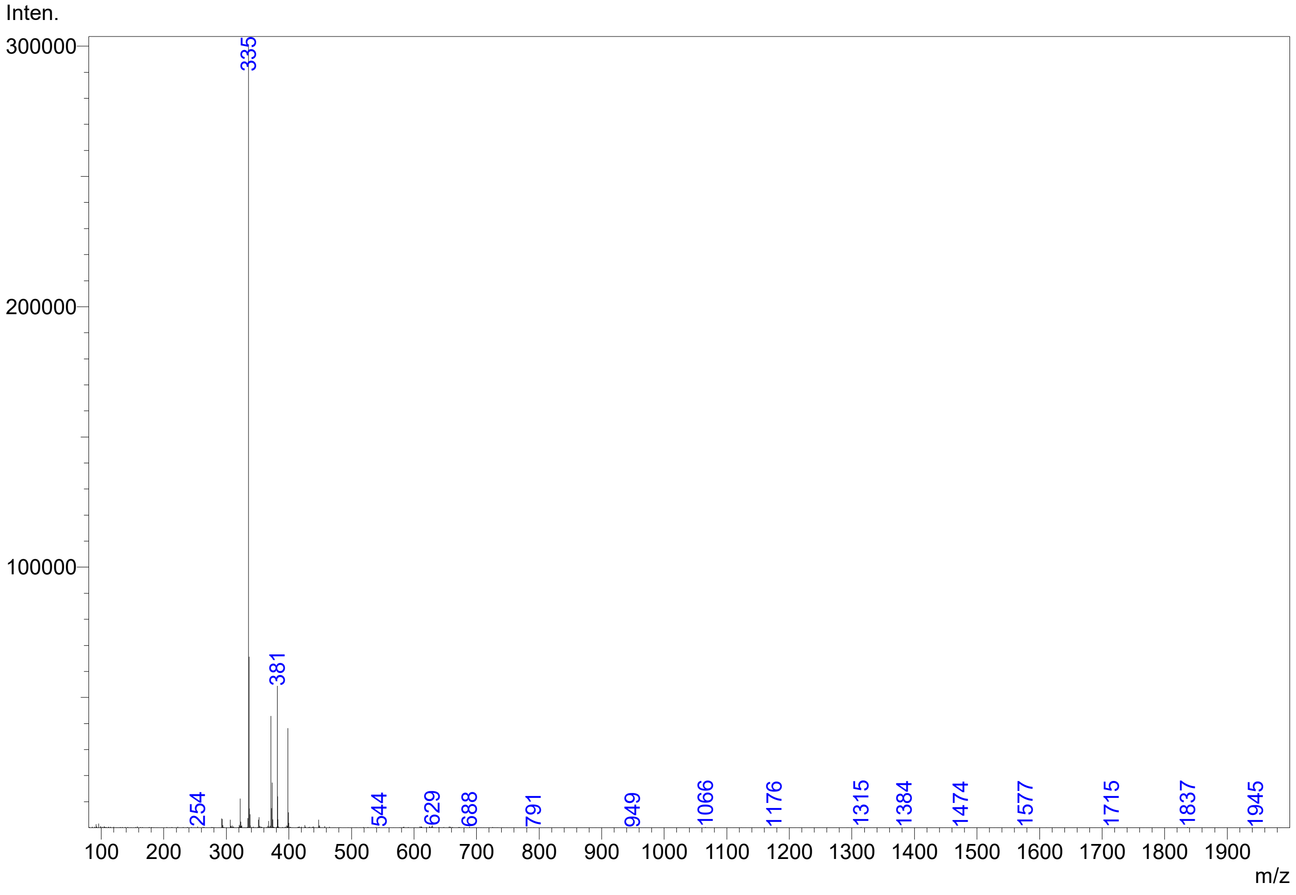

==== Shimadzu LabSolutions Browser Report ====

MS Spectrum(DO-142.lcd) Compound 10

Ret. Time: 1-1(E+) 1-2(E-) [0.347]

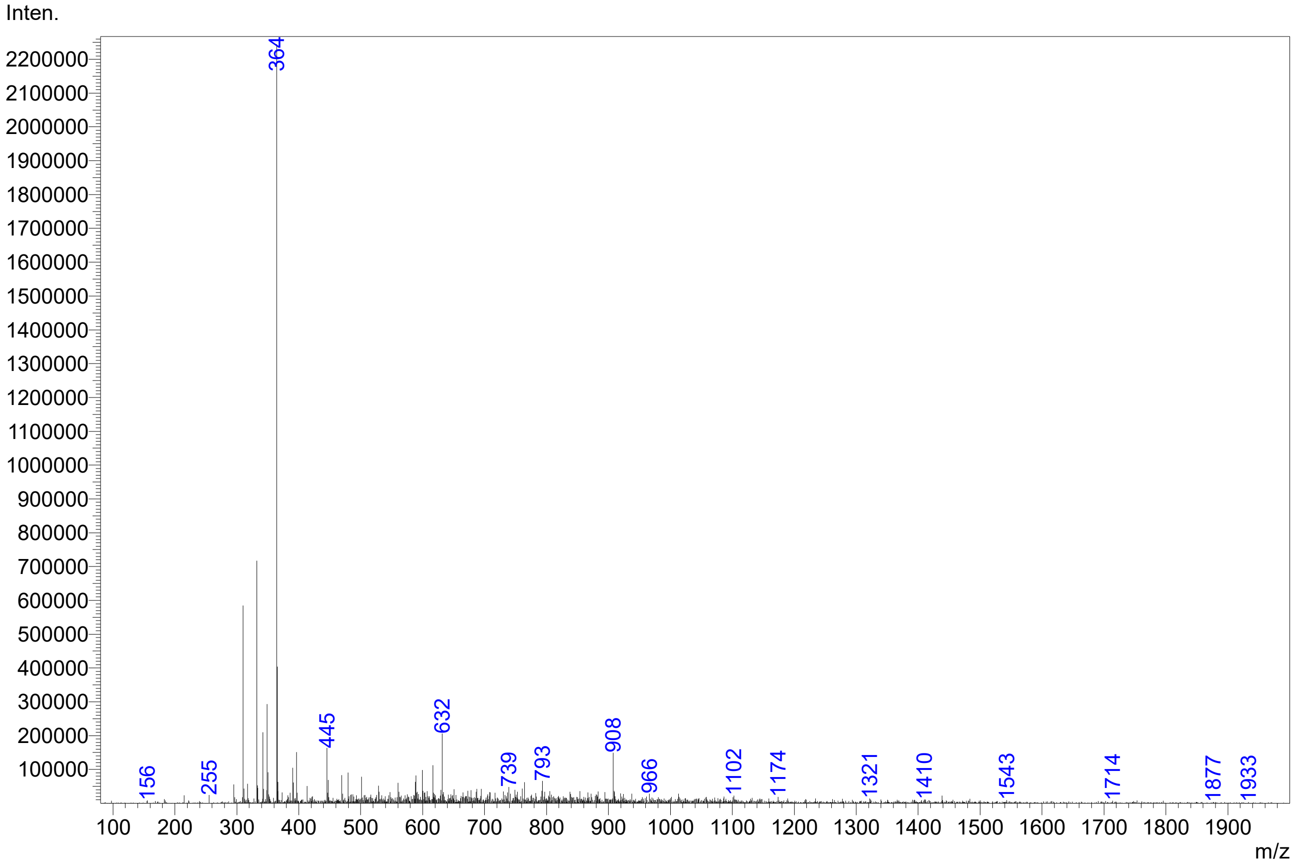

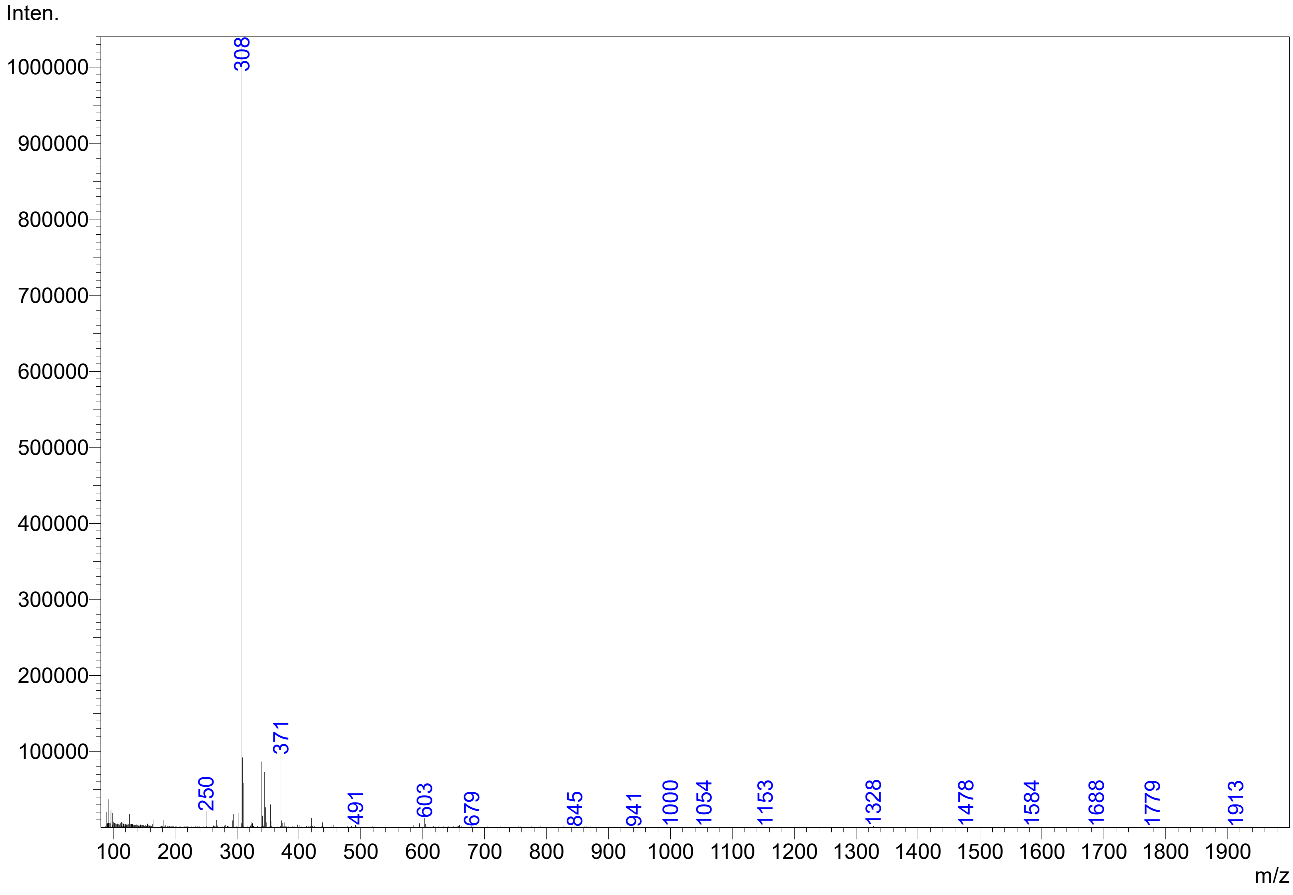

==== Shimadzu LabSolutions Browser Report ====

MS Spectrum(DO-166.lcd)

Compound 11

Ret. Time: 1-1(E+) 1-2(E-) [0.207->0.567]-[0.113->1.053]

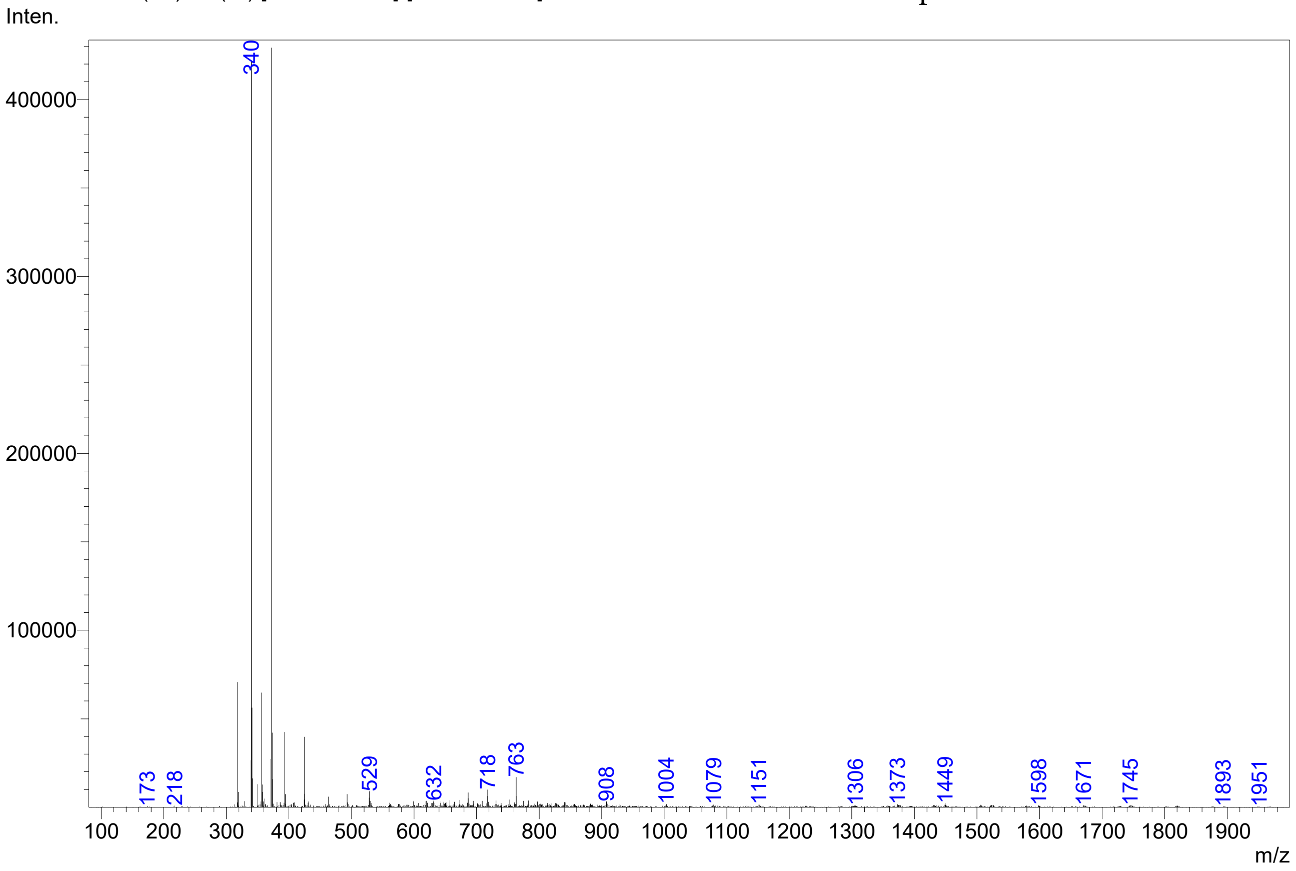

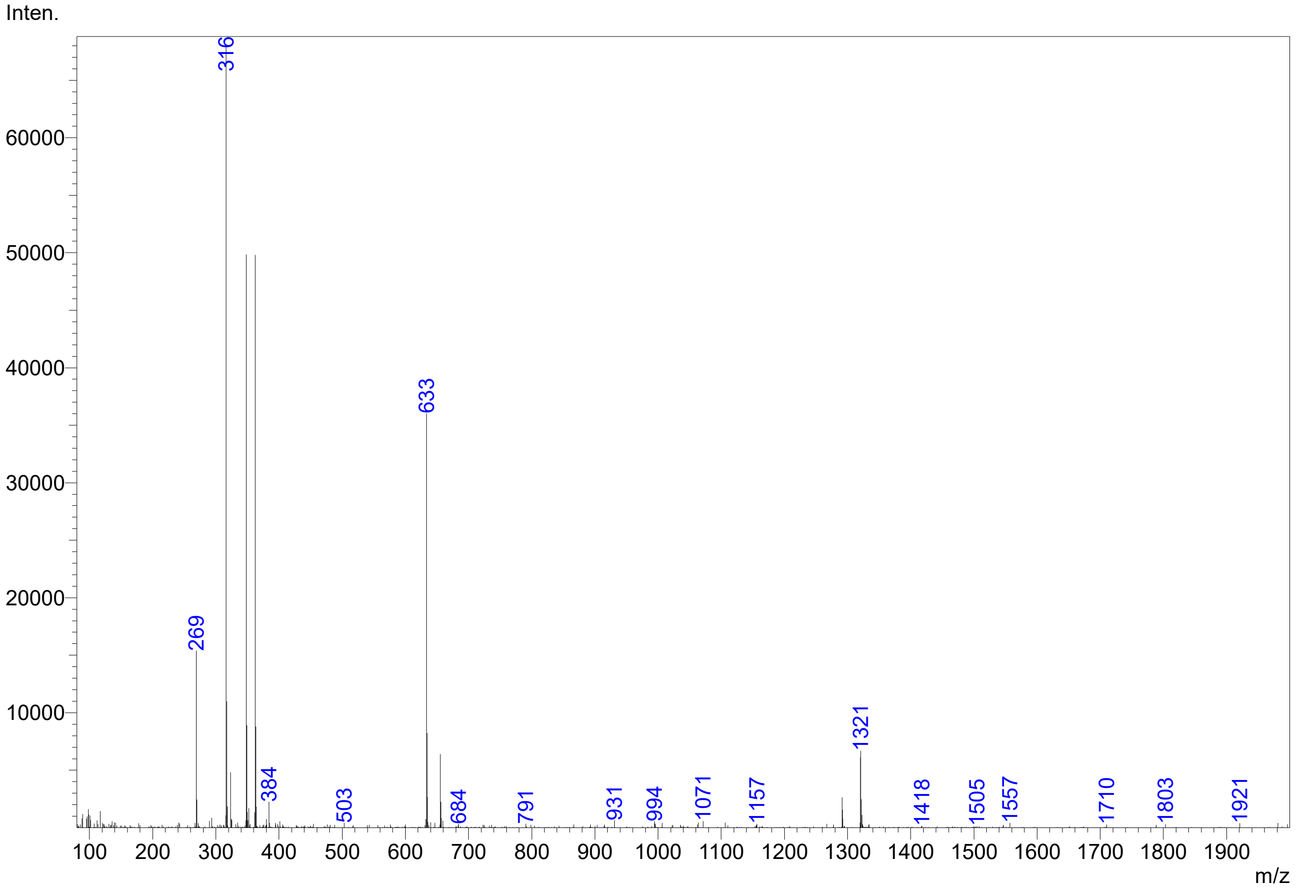

==== Shimadzu LabSolutions Browser Report ====

MS Spectrum(DO-190.lcd)

Ret. Time: 1-1(E+) 1-2(E-) [0.200->0.700]-[0.107->1.273]

Compound 12

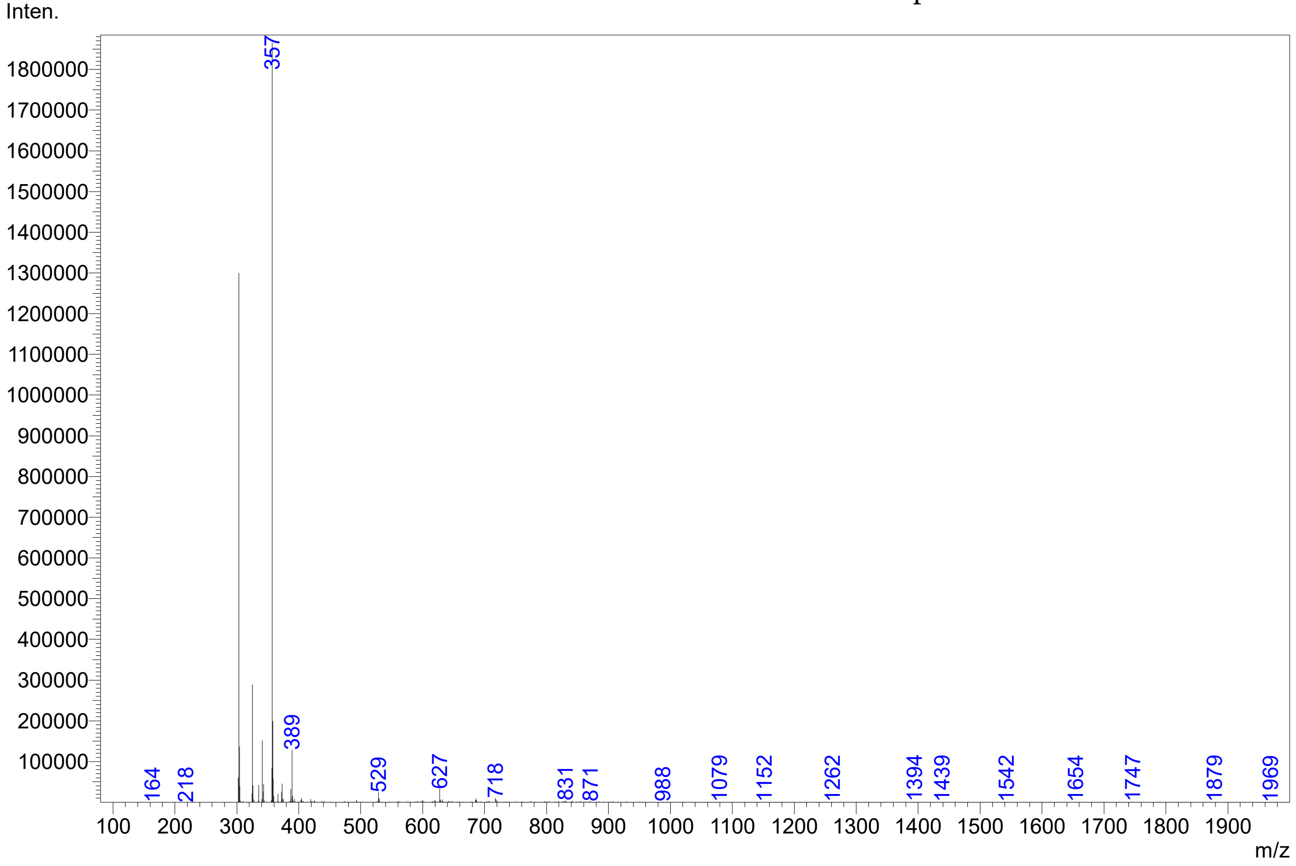

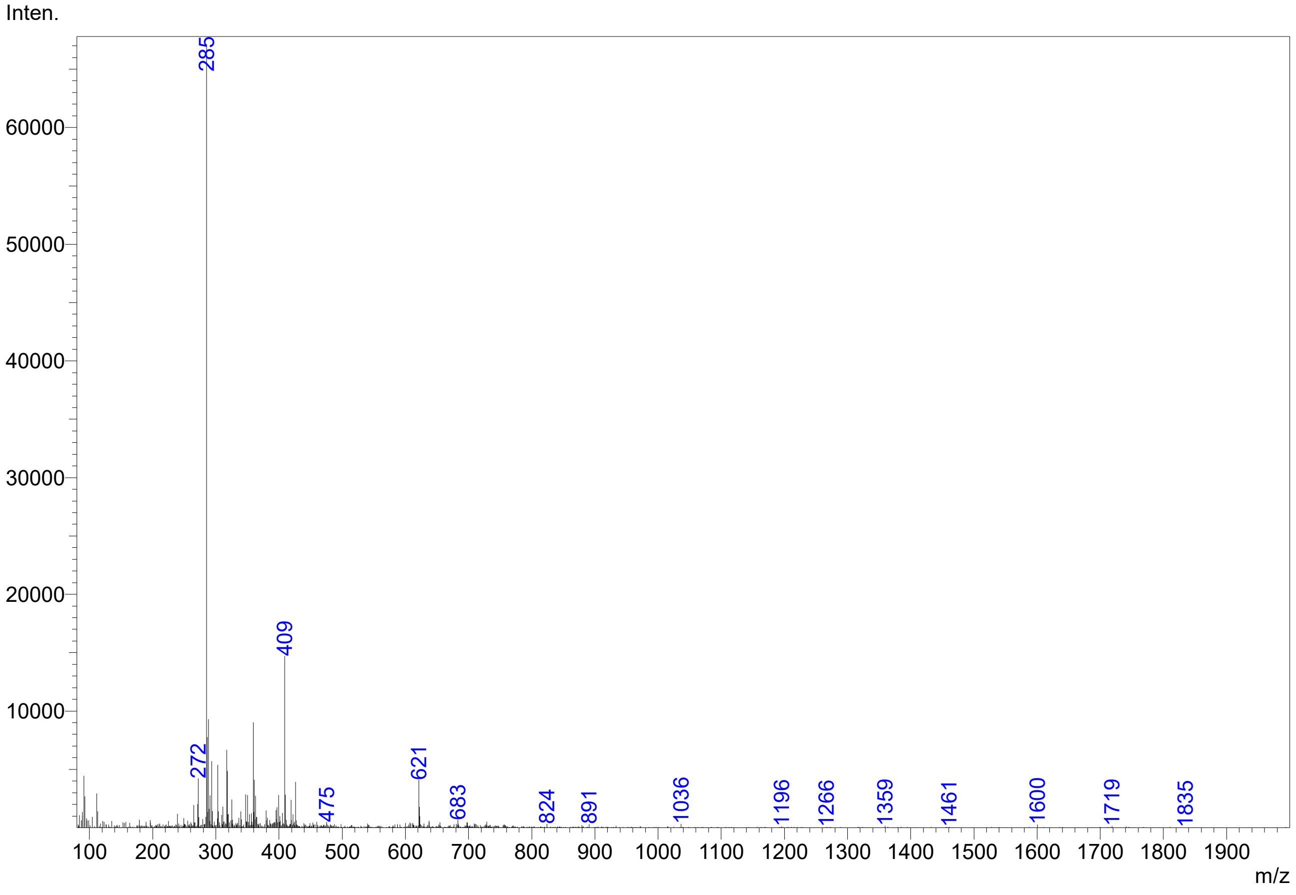

==== Shimadzu LabSolutions Browser Report ====

MS Spectrum(DO-209.lcd)

Compound 13

Ret. Time: 1-1(E+) 1-2(E-) [0.200->0.707]-[0.133->1.153]

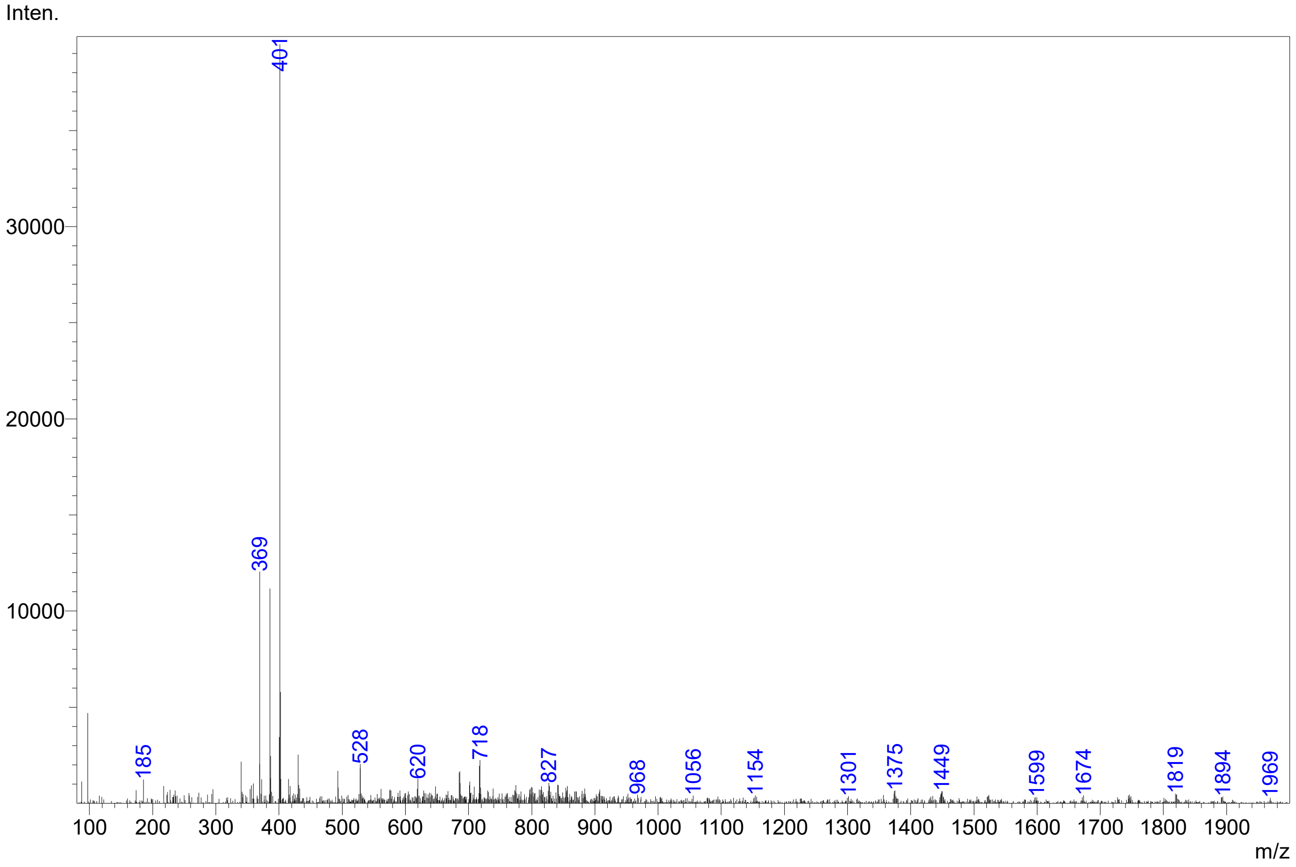

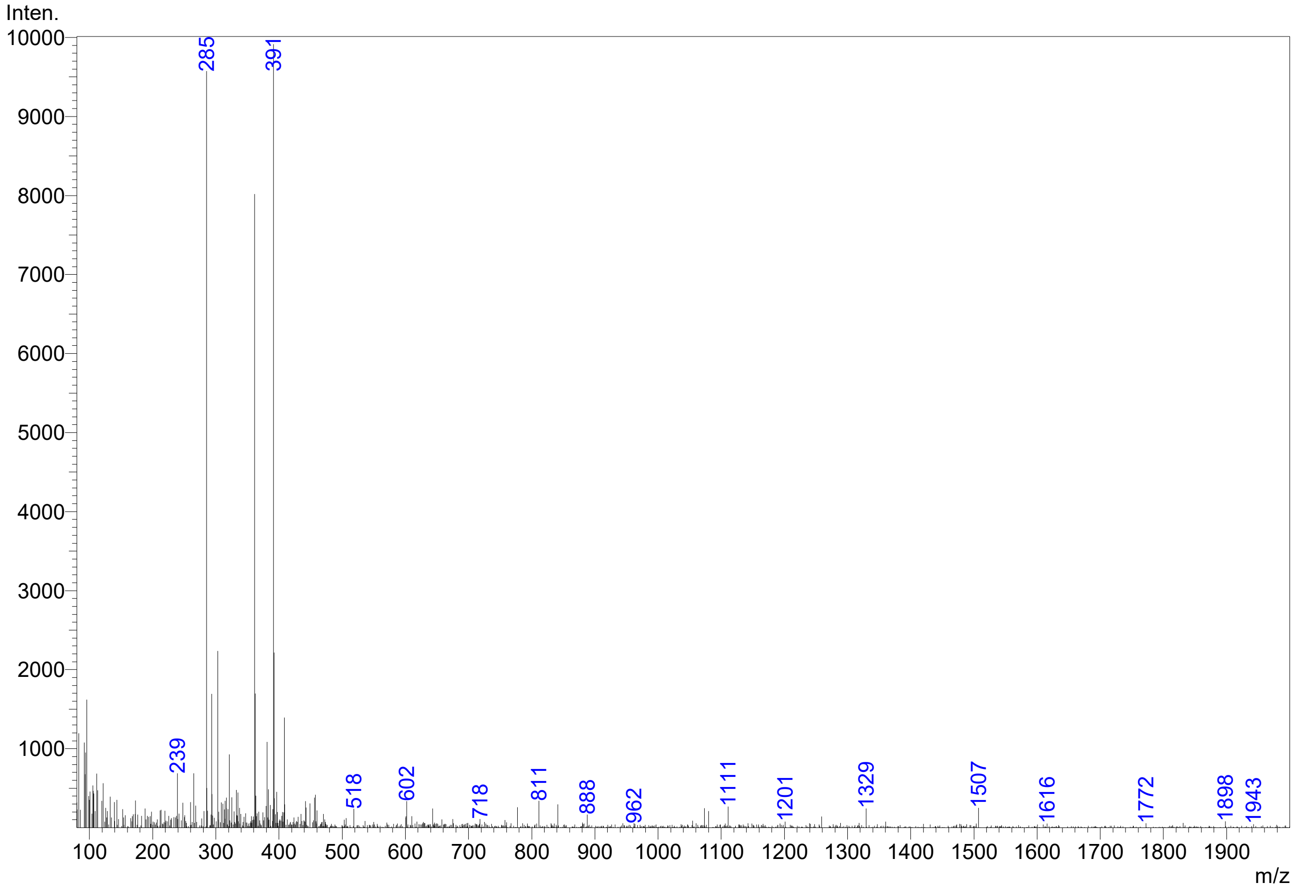

Supplement: Supplementary file 1 [file pharmaceuticals-15-01136-s001.zip › Supplementary material R1.pdf]
